# Delirium in older adults in the emergency department: a scoping review of current research and identified knowledge gaps

**DOI:** 10.1186/s12873-026-01582-z

**Published:** 2026-04-18

**Authors:** Siri Lerstøl Olsen, Marte Johanne Tangeraas Hansen, Elin Bø Lunde, Mathias Nikolai Petersen Hella, Anne Katrine Bergland

**Affiliations:** 1 Stavanger University Hospital Emergency Department Research Group, Stavanger, Norway; 2 SHARE – Centre for Resilience in Healthcare, Faculty of Health Sciences, University of Stavanger, Stavanger, Norway; 3 Research Group of Nursing and Healthcare Sciences, Stavanger University Hospital, Stavanger, Norway; 4 Department of Clinical Medicine, University of Bergen, Bergen, Norway; 5 Centre for Age-Related Medicine, Stavanger University Hospital, Stavanger, Norway; 6 Department of Clinical Sciences, University of Bergen, Bergen, Norway

**Keywords:** Delirium, Older patients, Geriatric patients, Emergency department, Prevention, Occurrence, Risk factors, Detection, Management, Consequences

## Abstract

**Background:**

Delirium is a common neurocognitive syndrome among older adults, associated with increased morbidity, mortality, and healthcare costs. Emergency departments (EDs) represent a critical point for early detection and prevention, yet delirium often remains under-recognized in this setting. Contributing factors include overcrowding, sensory overload, and lack of orientation aids, combined with demographic shifts leading to more acutely admitted older adults with complex needs. This scoping review aimed to map existing research on delirium in elderly patients in EDs and identify knowledge gaps.

**Methods:**

The review followed Joanna Briggs Institute methodology and adhered to the Preferred Reporting Items for Systematic reviews and Meta-Analyses extension for Scoping Reviews. Structured searches were conducted in EMBASE, MEDLINE, CINAHL, Web of Science, Cochrane, and PsycINFO for studies published between 2015 and July 2024, updated in September 2025. After duplicate removal, two reviewers independently screened 1,455 titles and abstracts, applying refined inclusion and exclusion criteria. 178 publications were reviewed in the research group. A total of 114 publications were included for full-text analysis.

**Results:**

Publications represented all continents, except for Africa, and most originated from North America (*n* = 54) and Europe (*n* = 35). Through content analysis, studies were categorized into six themes: Prevention, Occurrence, Risk factors, Detection, Management, and Consequences. Occurrence (*n* = 55) and Detection (*n* = 47) were the largest categories, while Prevention was least represented (*n* = 10). Evidence indicates wide variability in reported prevalence, inconsistent screening practices, and limited intervention research. Key gaps include standardized screening protocols, staff training, and effective prevention and management strategies tailored to ED environments.

**Conclusions:**

Delirium in older adults remains a significant challenge in EDs worldwide. This review synthesises current research on occurrence, risk factors, detection, and clinical outcomes, while highlighting critical gaps in strategies for prevention and management to secure optimal care. Despite valuable insights, evidence on effective prevention and the use of described toolkits in practice is limited. These gaps underscore the urgent need for targeted interventions and robust implementation research to improve recognition and care for this vulnerable patient population.

**Supplementary Information:**

The online version contains supplementary material available at 10.1186/s12873-026-01582-z.

## Background

Delirium is a neurocognitive syndrome characterised by acute cognitive decline, disturbed attention and altered awareness, ranging from a hypoactive state to hypervigilance and agitation [1, 2]. It is highly prevalent among hospitalised older adults, with the highest occurrence in critically ill patients [1]. A recent meta-analysis reported a delirium prevalence of approximately 23% among medical inpatients, with rates remaining relatively stable over time [3]. Despite its association with serious adverse outcomes—including increased morbidity, mortality, and prolonged hospitalisation—delirium frequently remains unrecognized [4]. Prevention, recognition and treatment of delirium continue to represent significant global healthcare challenges [1].

The Emergency Department (ED) represents a hospital entry point for a large volume of patients and thus an opportunity for delirium detection and prevention [4]. Demographic shifts, particularly the rising proportion of individuals over 65 years of age [5], are contributing to a higher demand for acute care among older adults [6, 7]. Consequently, EDs are seeing a growing number of patients with chronic illnesses, cognitive impairment, and geriatric syndromes such as delirium. Worldwide, EDs are increasingly challenged by overcrowding- a growing mismatch between patient needs and available staffing necessary to provide emergency care [8, 9]. Like other geriatric syndromes, delirium is often deprioritised in this busy ED setting [10]. Older patients might be delirious when admitted to the ED, or they might develop delirium in the ED setting (delirium incidence). EDs globally also experience ED boarding; a long waiting time for admitted patients in the ED before a bed is available in the hospital [11] prolonging the time older patients must stay in the ED. Factors in the ED that potentially contribute and enhances delirium includes sensory overload, disturbance of sleep, timing of medication- and nutrition administration, unfamiliar persons and lack of orientation items such as visual and hearing aids and clocks [12].

Given the association between delirium and serious complications, high use of hospital resources, and estimated significant healthcare costs [13], knowledge on how to effectively detect and manage this serious condition in the ED is warranted.

The objective of this scoping review is to map and summarise the existing body of research exploring various aspects of delirium in older patients in the ED setting and identify potential research gaps.

## Methods

This scoping review was conducted following the JBI Evidence synthesis paper by Pollock et al. [14] and the Preferred Reporting Items for Systematic reviews and Meta-Analyses extension for Scoping Reviews (PRISMA -ScR-checklist) [15]. A review protocol has not been registered.

The author group consisted of a carefully planned combination of academic doctors and nurses with collective expertise in geriatric medicine, emergency medicine, and nursing care skills for older patients who either have or are at risk of developing delirium.

### Search protocol and eligibility criteria

Literature search strategies were developed in collaboration with an expert librarian. We conducted the first online searches in July 2024, in: EMBASE, Epistemonikos, MEDLINE, CINAHL, Web of Science, Cochrane database and Psychinfo, for the period of 2000-july 2024 with the search terms: ‘delirium’ ‘sudden confusion, ‘sudden disorientation’ sudden psychosis/ ‘emergency- department or ward or room. The search was updated 15.09.2025 (Supplementary file A).

### Study selection

Our search found 2054 publications, which were uploaded into Rayyan (Rayyan: AI-Powered Systematic Review Management Platform). After removal of duplications, AKB and SLO separately screened the title and abstract of 1406 publications. During this process we refined our inclusion and exclusion criteria, deciding to exclude publications published before 2015 to increase the focus on the publications from the last decade. Then AKB and SLO discussed all publications with initial disagreement or uncertainty until a consensus was reached, or the publications were included in the next step with discussion in the research group. We excluded conference abstracts, published protocols, theses and literature summaries without research methodology. This was done due to the large amount of peer reviewed publications identified in the literature search. A total of 178 publications were included for full text reading and discussion using the recommended team approach [14] involving all co- authors in the process. We met regularly throughout the inclusion period to discuss the different publications, refine inclusion and exclusion criteria and come to an agreement on which publications to include. Certain articles were only partially included; specifically, only the sections pertaining to the emergency department were incorporated into our analysis. The team finally included 114 publications in the review.

#### Inclusion criteria:

- Publications from January 1, 2015, to September 25, 2025
- Languages: English, Norwegian, Danish, Swedish
- Publications with focus on older ED patients
- ED setting, including ED beds/ED- observation ward

### Data extraction

The process of data extractions involved familiarisations with the included publications. We created an extraction table to structure which information and results to extract from the publications (Table 1). All co- authors were included in piloting and customising the outline of the table for this specific review. The included publications in the extraction table were first reviewed by at least two researchers to ensure accurate content and secondly discussed in team meetings to make sure necessary information to answer the research questions was included.

**Table 1:** Overview over included publications. (* part of publication related to emergency department included)

Author	Year	Categories	Title	Country	Aim	Study type	Study description	Age (years)	Time to inclusion	Occurrence data (Screening tool)
Arendts et al.	2017	Detection	Rates of Delirium Diagnosis Do Not Improve with Emergency Risk Screening	Australia	To determine whether a bundled risk screening and warning or action card system improves formal delirium diagnosis and person-centered outcomes in hospitalized older adults	Quantitative	Prospective three phase trail. Using (ICD-10) delirium diagnostic criteria.	≥65	N.A.	Not reported
Arneson et al.	2023	Occurrence, Consequences	Association of delirium with increased short-term mortality among older emergency department patients: A cohort study	USA	To evaluate the association between delirium and subsequent short-term mortality in geriatric patients presenting to the ED	Quantitative	observational cohort study	≥75	Not reported	Prevalence: 11% DTS and bCAM
*Aslaner et al.	2017	Occurrence	Etiologies and delirium rates of elderly ED patients with acutely altered mental status: a multicenter prospective study	Turkey	To identify the etiologies of acute AMS (altered mental status), previous AMS histories, and coexistence of delirium in older patients to gain a better understanding of AMS and thus more properly manage elderly patients	Quantitative	Prospective observational study	≥65	Not reported	Prevalence: 55,7% in subgroup (bCAM)
Assis et al.	2022	Management	Modified Hospital Elder Life Program in the emergency department of a public university hospital: a multicomponent intervention program for preventing delirium	Brazil	To evaluate the feasibility of implementing an adaptation of the Hospital Elder Life Program (HELP) with the participation of family caregivers in a public university hospital	Multimethod	Exploratory descriptive pilot study	≥60	N.A.	N.A
Badawy & Shaban	2025	Management	Strategies to improve communication with older adults experiencing delirium in emergency departments: A systematic review	N.A	To identify barriers and enablers to communication with this population and synthesise evidence on effective strategies that support both communication and delirium management.	Review	Systematic review	N.A	N.A.	N.A.
Baten et al.	2018	Occurrence, Detection	Validation of the Brief Confusion Assessment Method for Screening Delirium in Elderly Medical Patients in a German Emergency Department	Germany	To evaluate the bCAM for a German hospital	Quantitative	Prospective observational cohort study.	≥70	within 12 hours	Prevalence: 16% (DSM-V)
Bonfichi et al.	2023	Prevention, Risk factors, Detection, Management	A Lethal Combination of Delirium and Overcrowding in the Emergency department	N.A	To analyze the risk factors associated with the occurrence of delirium in older ED patients and methods developed to screen, manage, and treat this condition.	Review	Narrative review	N.A.	N.A.	N.A.
Boucher et al.	2019	Occurrence, Detection, Consequences	Unrecognized incident delirium in older emergency department patients	Canada	To identify proportion of unrecognized incident ED delirium. Secondary: compare the two groups to assess the impact of unrecognized delirium	Quantitative	Sub analysis of multi- centre prospective cohort	≥65	<8 hours	Incidence within 24 h of ward admission: 10,43% (CAM)
Brich et al.	2019	Occurrence, Detection	Detecting delirium in elderly medical emergency patients: validation and subsequent modifcation of the German Nursing Delirium Screening Scale	Germany	To investigate the diagnostic performance of the German version of the Nu-DESC for delirium screening in an interdisciplinary German ED	Quantitative	Prospective observational study	≥70	<12 hours	Prevalence: 14.9% (DSM-V)
Butcher et al.	2022	Management	Utility of head computed tomography for older adults with suspected delirium in the emergency department: A retrospective observational study	Canada	To describe the utility of CT-head in older patients presenting to the ED with symptoms of delirium	Quantitative	Retrospective observational study	≥65	N.A.	N-A.
Buterakos & Keiser	2021	Detection, Management	Implementing a Delirium Admission Protocol in the Emergency Department	USA	To determine whether implementing a delirium screening for older adults in the ED with the bCAM and providing interventions to those who screen positive for delirium would decrease their hospital LOS and/or influence their discharge disposition	Other	Quality improvement study. Implementing BCAM in ED	≥65	N.A.	N.A.
Bédard et al.	2019	Occurrence, Detection	Validation of the O3DY French version (O3DY-F) for the screening of cognitive impairment in community seniors in the emergency department	Canada	To validate the Ottawa 3DY French (O3DY-F) Scale as a screening tool for delirium and cognitive impairment in a French speaking cohort	Quantitative	multicenter prospective study	≥65	8 hours	Prevalence 6% (CAM)
Béland et al.	2021	Occurrence, Risk factors	Predictors of delirium in older patients at the emergency department: a prospective multicentre derivation study	Canada	To confirm predicting factors of developing delirium in older emergency department patients	Quantitative	prospective multicentre cohort study	≥65	≥8 hours	Incidense delirium 11% (CAM)
Carpenter et al.	2024	Detection	Delirium detection in the emergency department: A diagnostic accuracy meta-analysis of history, physical examination, laboratory tests, and screening instruments	N.A	To quantify the diagnostic accuracy of approaches to identify delirium	Review	Systematic review	≥60	N.A.	N.A.
Cereda et al.	2025	Consequences	Multidimensional Radiological Assessment of Delirium in the Emergency Department	Italy	We aimed to propose a simple, multiparametric scoring system to improve prognostic stratification and help guide care in elderly patients with delirium presenting to the emergency department, facilitating care pathways for an acute condition with long-term chronic implications.	Quantitative	A retrospective analysis of prospectively collected data from all patients consecutively admitted to OBI-GER during its first year of operation. OBI-GER is an Geriatric Short-Stay Observation Units (G-SSU) - within the emergency department (ED)	Mean 86.8	N.A.	N.A.
*Chary et al.	2025	Detection, Management	Emergency Nurses’ Perspectives on Adopting Geriatric Screenings for Cognitive Impairment: A Qualitative Study	USA	To use implementation science to understand ED nurses’ perspectives about implementing screenings for delirium and dementia.	Qualitative	Semi-structured qualitative interviews with ED nurses in an academic emergency department	N.A.	N.A.	N.A.
Chary et al.	2023	Detection	Leveraging the Electronic Health Record to Implement Emergency Department Delirium Screening	USA	To understand how EDs use health information technology, and specifically the electronic health records, to support implementation of delirium screening	Qualitative	Semi-structured interviews	N.A.	N.A.	N.A.
Chary et al.	2022a	Prevention	A Qualitative Study of Emergency Department Delirium Prevention Initiatives	USA	To characterize delirium prevention initiatives in EDs in the United States and Canada	Qualitative	Individual interviews were conducted using an online audio-visual platform	N.A.	N.A.	N.A.
Chary et al.	2021	Detection, Management	A Survey of Delirium Self-Reported Knowledge and Practices Among Emergency Physicians in the United States	USA	To provide a national survey-based preliminary assessment of emergency physicians’ knowledge, attitudes, and practices about ED delirium in the United States	Quantitative	An electronic survey	N.A.	N.A.	N.A.
Chary et al.	2024	Detection	Implementation of delirium screening in the emergency department: A qualitative study with early adopters	USA and Canada	This study aimed to understand system-level experiences of EDs that successfully adopted delirium screening.	Qualitative	Individual interviews	N.A.	N.A.	N.A.
Chary et al.	2022b	Detection	Language Discordance in Emergency Department Delirium Screening: Results from a Qualitative Interview-Based Study	USA and Canada	To determine current practices and identify concerns about ED delirium screening in situations of language discordance among early adopter EDs	Qualitative	Semi-structured qualitative interviews in 20 Eds	N.A.	N.A.	N.A.
Choutko-Joaquim et al.	2019	Occurrence, Risk factors	Associations between Frailty and Delirium among Older Patients Admitted to an Emergency Department	Switzerland	To explore the relationships between frailty and delirium in older adult patients consulting at an emergency department (ED) in Switzerland, and secondary to analyze associations between frailty and delirium among those older adults during the 4 h following their admission to an ED	Quantitative	Cross-sectional	N.A.	N.A.	Prevalence 2% (CAM) 12% subdromal delirium (CAM)
Cirbus et al.	2019	Risk factors, Consequences	Delirium ethology subtypes and their effect on six-month function and cognition in older emergency department patients	USA	To explore how delirium subtyped by ethology affected six-month function and cognition	Quantitative	Pre-planned secondary analysis of a prospective study.	N.A.	N.A.	N.A.
Daoust et al.	2020	Occurrence, Risk factors	Relationship Between Pain, Opioid Treatment, and Delirium in Older Emergency Department Patients	Canada	To assess the relationship between pain, opioid treatment, and delirium in older ED patients	Quantitative	Prospective cohort study (multicentre)	N.A.	N.A.	Incidence: Delirium after 8 hours and before 24 h = 12%. (CAM)
*Demirtakan et al.	2023	Occurrence, Consequences	Clinical assessment and short-term mortality of older adults using RASS and 4AT tools	Turkey	To reveal and compare the clinical outcomes and etiologic factors of older patients with delirium, stupor and coma. The secondary objective was to identify the 30-day mortality risk for those patients.	Quantitative	Prospective observational study	N.A.	N.A.	Prevalence: 40.6% in subgroup (4AT)
Dogan et al.	2022	Consequences	CRP/albumin, Glasgow prognostic score, and prognostic nutritional index as a predictor of mortality among delirium patients	Turkey	To analyze the parameters that could predict 30 days of mortality of the patients diagnosed in the emergency department (ED) with delirium	Quantitative	Retrospective patient record study	N.A.	N.A.	N.A.
*Dyer et al.	2016	Occurrence, Management	Cognitive assessment of older adults at the acute care interface: the informant history	Ireland	Assess the informant history in the complete cognitive assessment of older adults	Quantitative	Using CAM-ICU for delirium (and MMSE andAD8 for dementia)	N.A.	N.A.	Prevalence 11.8% CAM-ICU
Eagles et al.	2022	Detection	Barriers and facilitators to nursing delirium screening in older emergency patients: a qualitative study using the theoretical domains framework	Canada	To identify barriers and facilitators to delirium screening by nurses in older ED patients	Qualitative	Semi-structured interviews	N.A.	N.A.	N.A.
Ehrlich et al.	2024	Prevention	Managing Delirium in the Emergency Department: An Updated Narrative Review	USA	To describe the components of successful delirium management strategies to be used by EDs in building delirium management programs	Review	Narrative Review	≥65	N.A.	N.A.
Elder et al.	2023	Risk factors	Emergency Department Length of Stay Is Associated with Delirium in Older Adults	USA	To evaluate the association between development of incident delirium during admission and (a) ED LOS, (b) ED hallway time, and (c) number of times a patient is moved from one treatment space to another for non-clinical reasons within the ED	Quantitative	Retrospective cohort study	≥65	N.A.	N.A.
Elsayem et al.	2017	Consequences	Advance Directives, Hospitalization, and Survival Among Advanced Cancer Patients with Delirium Presenting to the Emergency Department: A Prospective Study	USA	To determine the frequency of delirium in these patients. Our secondary aim was to compare hospital admission rate, ICU admission rate, presence of advance directives, hospital mortality, and overall survival between the patients with and without delirium.	Quantitative	A prospective cross-sectional observational study	Median age 62	<12 hours	Prevalence 9% (CAM and MDAS)
Elsayem et al.	2016	Occurrence	Delirium frequency Among advanced Cancer patients presenting to an emergency department: a prospective randomized observational study	USA	To determine delirium frequency and recognition by ED physicians among patients with advanced cancer presenting to the ED of The University of Texas MD Anderson Cancer Center. We also determined the accuracy of ED physician assessments of delirium	Quantitative	Prospective, cross-sectional, observational study.	Median age 62	≤12 hours	Prevalence in subgroup: 10% of patents ≥ 65 y (CAM)
*Émond et al.	2017	Occurrence, Risk factors, Consequences	Emergency Department Stay Associated Delirium in Older Patients	Canada	To assess the incidence and impacts of ED stay associated delirium	Quantitative	Record review	≥65	≥12 hours	ED related delirium: Incidence of 18% (Chart based CAM)
´Émond et al.	2018	Occurrence, Risk factors	Incidence of delirium in the Canadian emergency department and its consequences on hospital length of stay: a prospective observational multicentre cohort study	Canada	To determine the incidence of delirium and describe its impacts on hospital length of stay among non-delirious community-dwelling older adults with an 8-hour exposure to the emergency department (ED) environment.	Quantitative	A prospective observational multicentre cohort study (March–July 2015).	≥65	≥8 hours	The mean incidence of ED-related delirium was 12.1% (*n* = 41) (CAM)
*Evensen et al.	2018	Risk factors	Environmental factors and risk of delirium in geriatric patients: an observational study	Norway	To specifically investigate if ward-transfers, arriving ED at nighttime, time spent in ED and visits from other specialists and radiological procedures at nighttime (nighttime investigations) are associated with development of delirium (incident delirium) and delirium motor subtypes in patients acutely admitted to a geriatric ward	Quantitative	Prospective observational study	≥75	With in 24 hours	ED related delirium: Incidence of 19,3% (DSM-IV)
Evensen et al.	2019	Occurrence	Delirium and cognitive impairment among older patients in Norwegian emergency departments	Norway	To investigate the prevalence of delirium among elderly patients in Norwegian emergency departments on World Delirium Awareness Day, 14 March 2018	Quantitative	Point prevalence survey	≥75	Within 24 hours	Prevalence 17% (4AT)
Filiatreault et al.	2024	Management	Developing a set of emergency department performance measures to evaluate delirium care quality for older adults: a modifed e-Delphi study	Canada	To gain consensus on a set of quality statements and PMs that can be used to evaluate delirium care quality for older ED patients	Other	modified eDelphi	N.A.	N.A.	N.A.
*Filiatreault et al.	2023	Prevention, Detection, Management	A critical appraisal and recommendation synthesis of delirium clinical practice guidelines relevant to the care of older adults in the emergency department: An umbrella review	N.A	To appraise and synthesize clinical practice guidelines for delirium care relevant to older patients	Review	Umbrella review	N.A.	N.A.	N.A.
Fossen & Bergland	2023	Detection	Challenges with identifying delirium in elderly patients with hip fractures in the emergency room – a qualitative study of nurses’ experiences	Norway	To explore nurses’ experiences with challenges regarding identification of delirium in elderly patients with hip fractures in the emergency room	Qualitative	Qualitative explorative design, using semi-structured individual interviews	N.A.	N.A.	N.A.
Gagné et al.	2018	Occurrence, Detection	Performance of the French version of the 4AT for screening the elderly for delirium in the emergency department	France	To evaluate the performance of the French version of the 4 A’s Test (4AT-F) for the detection of delirium and cognitive impairment in older patients	Quantitative	Multicentre study. Comparing the French version of 4AT with the CAM and the Telephone Interview for Cognitive Status (TICS)s	≥65	>8 hours	Total occurence 15.3%. ED-related Incident delirium 10%. (using CAM) within 24 H
Giroux et al.	2018	Occurrence, Risk factors	Frailty Assessment to help predict patients at risk of delirium when consulting the emergency department	Canada	To assess if screening older patients for frailty in EDs could help identify those at risk of delirium	Quantitative	prospective cohort study	≥65	≥8 hours	Incident Delirium occurred in 28,6% (20/70) frail patients and in 20/265 (7.6%) non-frail- within 24 h of ward admission- (CAM)
Giroux et al.	2021	Occurrence, Risk factors	Functional and cognitive decline in older delirious adults after an emergency department visit	Canada	To evaluate the impact of emergency department (ED) stay-associated delirium on older patient’s functional and cognitive status at 60 days post ED visit	Quantitative	Multi-centre prospective cohort study in 5 Eds, this study was part the INDEED study.	≥65	>8 hours	11,3% of the 608 patients had delirium during their ED stay or within 24 hours of the ward admission. (CAM).
Goll et al.	2025	Occurrence, Risk factors	Frailty screening of older patients in emergency departments in Norway	Norway	To map the incidence of frailty in older patients in two emergency departments in Norway	Quantitative	Prospective quality improvement project.	≥75	Not reported	Prevalence:25% (with 4AT)
Grossman et al.	2017	Occurrence, Detection	Performance of the modified Richmond Agitation Sedation Scale in identifying delirium in older ED patients	Switzerland	To test the performance criteria of the modified Richmond Agitation Sedation Scale (mRASS) in identifying delirium in older ED patients.	Quantitative	This was a pre-planned subanalysis of data collected in a larger prospective trial in which we tested the performance of the modified Confusion Assessment Method for the ED	≥65	N.A.	Prevalence 7% (DSM-IV)
Han et al.	2015	Occurrence, Detection	The Diagnostic Performance of the Richmond Agitation Sedation Scale for Detecting Delirium in Older Emergency Department Patients	USA	To explore the diagnostic accuracy of the RASS for delirium in older ED patients	Quantitative	Prospective observational study	≥65	<12 hours	Prevalence 12.3% (DSM-IV)
*Han et al.	2018	Detection	An evaluation of single question delirium screening tools in older emergency department patients	USA	To evaluate diagnostic performance of several single question delirium screens both to patients and next of kin	Quantitative	Prospective observational study.	≥65	N.A.	Prevalence 12% (DSM IV)
Han et al.	2016	Occurrence, Detection	A quick and easy delirium assessment for non-physician research personnel	USA	To evaluate the diagnostic accuracy and reliability of the modified bCAM	Quantitative	Secondary analysis of prospective observational study: studying CAM-ICU), bCAM, and the Delirium Triage Screen in older ED patients.	≥65	<12 hours	Prevalence:12% (DSM IV)
Han et al.	2017 a	Occurrence, Consequences	Exploring Delirium’s Heterogeneity: Association Between Arousal Subtypes at Initial Presentation and 6-Month Mortality in Older Emergency Department Patients	USA	To determine if the arousal subtype of delirium (normal, decreased, and increased) is associated with 6-month mortality compared with patients without delirium.	Quantitative	A cross-sectional, observational retrospective study	≥65	<12 h	Prevalence 14,3% (CAM-ICU)
Han et al.	2017b	Consequences	Delirium in the Emergency Department and Its Extension into Hospitalization (DELINEATE) Study: Effect on 6-month Function and Cognition	USA	To describe the extent to which delirium in the ED persists into hospitalization (ED delirium duration) and to determine how ED delirium duration is associated with 6-month functional status and cognition	Quantitative	A prospective cohort study	≥65	0 hours	N.A.
Han et al.	2019	Risk factors	Supratherapeutic Psychotropic Drug Levels in the Emergency Department and Their Association with Delirium Duration: A Preliminary Study	USA	To determine the frequency of supratherapeutic psychotropic serum drug levels (SPDLs) in older hospitalized patients and if it is associated with emergency department (ED) delirium duration	Quantitative	Prospective cohort study (secondary analysis) from the cohort of patients in the DELINEATE study	≥65	<4hhours	N.A.
Hasemann et al.	2018	Occurrence, Detection	Screening and detection of delirium in older ED patients: performance of the modified Confusion Assessment Method for the Emergency Department (mCAMED). A twostep tool	Sveits	To investigate the performance criteria of the mCAM-ED tool in a consecutive sample of older ED patients and to evaluate the performance of the mCAM-ED in patients with and without dementia and to test whether this tool is efficient in keeping evaluation time to a minimum and reducing screening and assessment burden on the patient	Quantitative	Single center prospective validation study.	≥ 65	N.A.	Prevalence 7% (DSM-IV)
Hsieh et al.	2015	Occurrence, Consequences	Clinical deterioration in older adults with delirium during early hospitalisation: a prosepective cohort study	USA	Measure prevalence and incidence of delirium in older adults as they transit from the ED to the inpatient ward and to determine the association between delirium during early hospitalisation and subsequent clinical deterioration	Quantitative	Prospective cohort study. Assessed delirium from beginning of ED, daily for 3 days using CAM-ICU	≥65	N.A.	Prevalence 11% (CAM-ICU)
Hullick et al.	2018	Detection, Management	An assistant workforce to improve screening rates and quality of care for older patients in the emergency department: findings of a pre- post, mixed methods study	Australia	To evalulate changes in screening rate and care after use of assistant work force, OPTA (Older Person Technical Assistant)	Mixed Methods	Collection of data before and after introduction of OPTA and focus group interview	≥75	N.A	Nor reported
Israni et al.	2018	Consequences	Delirium as a predictor of mortality in US Medicare beneficiaries discharged from the emergency department: a national claims-level analysis up to 12 months	USA	To analyse mortality rates among seniors discharged from the ED with delirium up to 12 months at the national level	Quantitative	Retrospective analysis	≥65	N.A	N.A.
*Joseph et al.	2024	Risk factors	Boarding Duration in the Emergency Department and Inpatient Delirium and Severe Agitation	USA	To evaluate the association of ED boarding duration with inpatient delirium and agitation	Quantitative	Retrospective cohort study	Mean age 66.5	N.A.	delirium or agitation 11.5%
Keene et al.	2023	Prevention, Occurrence	Feasibility of Light and Music Therapy in the Elderly for the Prevention of Hospital-Associated Delirium	USA	To examine the effect of light and/or music on the incidence of hospital-associated delirium within the first 24 hours	Quantitative	Pilot RCT	≥65	N.A.	Incidence within 24 h in subgroup (CAM) (See main results)
Kennedy et al.	2021	Prevention, Detection, Management	ED-DEL: Development of a change package and toolkit for delirium in the emergency department	USA	To create a resource that can be disseminated widely and used to improve delirium identification, prevention, and management in older adults in the ED	Other	Quality improvement	N.A.	N.A.	N.A.
Lafuente-Lafuente et al.	2025	Risk factors	Development of a checklist for systematic screening of precipitating factors in older patients admitted to hospital with delirium	France	To develop a checklist to help clinicians screen for acute precipitants of delirium in older patients	Other	Modified Delphi study with a pilot trial	N.A.	N.A.	N.A.
LaMantia et al.	2017	Detection, Management	Emergency medical service, nursing, and physician providers’ perspectives on delirium identification and management	USA	To understand providers’ perceptions regarding identifying and treating older adults with delirium in the pre-hospital and emergency department environments	Qualitative	Focus group interviews	N.A.	N.A.	N.A.
Lee J. et al.	2022	Occurrence, Detection	Prevalence, management and outcomes of unrecognized delirium in a National Sample of 1,493 older emergency department patients: how many were sent home and what happened to them?	Canada	To prospectively establish the rate of delirium recognition by ED physicians and nurses in a multi-centre sample of older ED patients. Specifically, our objectives were to measure: (i) delirium recognition by nurses and physicians, (ii) confidence of each profession in their diagnosis of delirium, (iii) intended disposition (admit or discharge location), (iv) confidence of each profession in their intended disposition destination (v) actual disposition and (vi) outcomes of patients with unrecognized delirium	Quantitative	Prospective Observational Study	≥65	<4 hours	Prevalence 5.3% (CAM)
*Lee S. et al.	2023	Management	Delirium, confusion, or altered mental status as a risk for abnormal head CT in older adults in the emergency department: A systematic review and meta-analysis	N.A	To report frequency of abnormal findings in CT in patients with abnormal mental status in the ED.	Review	Systematic review	≥65	N.A.	N.A.
*Lee S. et al.	2022**b**	Occurrence, Risk factors	Opioid and benzodiazepine use in the emergency department and the recognition of delirium within the first 24 hours of hospitalization	USA	To test the hypothesis that opioids and benzodiazepines exposure in the emergency department (ED) is associated with delirium	Quantitative	Retrospective cohort study	≥65	N.A	Prevalence 11.1% (ICD-9 or 10)
Lee S. et al.	2018	Occurrence, Detection	The point-of-care EEG for delirium detection in the emergency department	USA	To pilot BSEEG (bispectral EEG) to diagnose delirium in the ED	Quantitative	Prospective pilot study	≥65	<8 hours	Prevalence 18,8% (using clinical impression/CAM-ICU or DRS-98 > 18).
Lee S. et al.	2022a	Prevention, Management	Can we improve delirium prevention and treatment in the emergency department? A systematic review	N.A	To evaluate any interventions to prevent incident delirium, or shorten the duration of prevalent delirium, in older adults presenting to the emergency department	Review	Systematic Review	≥65	N.A.	N.A.
Lucke et al.	2019	Occurrence, Detection	CAM-ICU may not be the optimal screening tool for early delirium screening in older emergency department patients: a prospective cohort study	Netherlands	To investigate the prevalence of delirium in two EDs in the Netherlands by using the CAM-ICU and 6-CIT, performed within 1 h after arrival to the ED	Quantitative	Prospective cohort study	≥70	<1 hours	1.3% CAM-ICU/ 9.3% 6-CIT
Ma et al.	2018	Consequences	Increased Readmission Risk and Healthcare Cost for Delirium Patients without Immediate Hospitalization in the Emergency Department	Korea	To explore whether (i) immediate hospitalization influences the readmission risk of patients with delirium; (ii) the readmission risk is affected by various risk factors; and (iii) the healthcare cost differs between groups within 28 days of the first ED visit.	Quantitative	Using the National Health Insurance Research Database, the data of 2,780 subjects presenting with delirium at an ED visit from 2000 to 2008 were examined.	≥65	N.A.	N.A.
MacLullich et al.	2019	Detection	The 4 ‘A’s test for detecting delirium in acute medical patients: a diagnostic accuracy study	UK	To evaluate 4ATs usability, diagnostic accuracy and cost.	Multimethod	Two phases: Surveys, interviews and observations with healthcare personnel- including the ED. 2. Diagnostic accuracy study comparing 4AT with CAM. In addition, an Economic evaluation.	>70	N.A.	Prevalence 12.1% (by reference standard, DRS98, DSM-IV and a consensus)
Mailhot et al.	2021	Detection, Managment	An emergency department delirium screening and management initiative: the development and refinement of the SCREENED-ED Intervention.	Canada	To adress challenges of managing delirium in the ED, an intervention was aimed at ED nurses and physicians.	Other	Quality improvement	N.A.	N.A.	N.A.
Mailhot et al.	2020	Occurrence, Detection, Consequences	Family Identification of Delirium in the Emergency Department in Patients With and Without Dementia: Validity of the Family Confusion Assessment Method (FAM-CAM)	USA	To examine the ability of the family-rated Family Confusion Assessment Method (FAM-CAM) to identify delirium in the emergency department (ED) among patients with and without dementia.	Quantitative	Prospective validation study	≥70	N.A.	Prevalence: 28% (CAM-ICU)
*Marcomini et al.	2024	Occurrence, Risk factors	Evaluation of Delirium Among Elders in the Emergency Department: A Cross-Sectional Study	Italy	To evaluate the prevalence of risk of delirium in people 65 years or older hospitalized in the ED for a minimum of 8 hours and within 24 hours of attendance. The study’s secondary goal was to identify the variables that influenced the risk of delirium.	Quantitative	Cross sectional survey (2 EDs)	≥65	>8 h < 24 h	Prevalence 29% (4AT)
Marra et al.	2018	Occurrence, Detection	Focusing on Inattention: The Diagnostic Accuracy of Brief Measures of Inattention for Detecting Delirium	USA	To evaluate the diagnostic performance of of reciting the months of year backwards (MOTYB) from December to July (MOTYB-6) and December to January (MOTYB-12) for delirium as diagnosed by a reference standard. We also explored other brief tests of inattention such as spelling a word (“LUNCH”) backwards, reciting the days of the week backwards, 10-letter vigilance “A” task, and five-picture recognition task	Quantitative	Preplanned secondary analysis of a prospective observational study.	≥65	<12 h	Prevalence: 10,6% delirium (DSM-IV)
Martin et al.	2022	Detection	Implementing delirium screening in the emergency department: a quality improvement project	Ireland	To apply Lean six sigma tools and methodology to increase the number of older adults (≥65 years) being screened for delirium using the 4AT tool. The goal was that 70% of patients would be screened for delirium at the end of the project	Other	Quality improvement	≥65	N.A.	N.A.
McNeil et al.	2019	Risk factors	Plasma biomarkers of inflammation, coagulation, and brain injury as predictors of delirium duration in older hospitalized patients	Italy	To determine if plasma biomarkers of inflammation (interleukins 6 and 8, soluble tumour necrosis factor receptor I, coagulation (Protein C), endothelial activation (plasminogen activating inhibitor-1 [PAI-1]), and BBB injury (S100B) were associated with ED delirium duration in older ED patients admitted to the hospital	Quantitative	Prospective cohort study	≥65	<4 h	N.A.
Minion et al.	2021	Detection	An educational module to improve knowledge of delirium screening in the Emergency Department	USA	To increase HCP knowledge and use of bCAM	Quantitative	Pre-post	N.A.	N.A.	N.A.
Miró et al.	2024	Occurrence, Risk factors	Hyperactive delirium during emergency department stay: analysis of risk factors and association with short term outcomes	Spain	To investigate factors related to the development of hyperactive delirium in patients during emergency department (ED) stay and the association with short-term outcomes.	Quantitative	Multicenter cohort study	≥65	N.A.	Incidence 1.6% of hyperactive delirium (requiring neuroleptics or benzodiazepines)
Morales- Puerto et al.	2024	Occurrence, Detection	Spanish transcultural adaptation of the 4AT score for the evaluation of delirium in the emergency department: a prospective diagnostic test accuracy study	Spain	To adapt the 4AT to the Spanish language and evaluate its validity for detecting delirium in patients admitted to the ED of the Hospital Costa del Sol, Marbella (Spain)	Quantitative	Prospective diagnostic accuracy study	≥65	>4 h	Prevalence 6.3% using DRS-R98.
Mota et al.	2016	Occurrence, Risk factors	Association of education with occurrence of delirium	Brazil	To determine the ED prevalence of delirium and its association with education in all 18 years or older patients.	Quantitative	Cross- sectional study.	43,6% >70	Not reported	>70 years of age: 43,6% Delirium (CAM)
Ngyuen et al.	2017	Occurrence, Risk factors	The Delirium Drug Scale is associated to delirium incidence in the emergency department	Canada	To validate the association between the DDS score and the incidence of delirium	Quantitative	Observational retrospective cross-sectional chart review study	≥75	N.A.	Prevalence of delirium (within 48 h of arrival) 19.1% (CHART REVIEW)
Ni Chróinín et al.	2025	Prevention, Management	A pilot trial exploring the use of music in the emergency department and its association with delirium and other clinical outcomes	Australia	To assess potential feasibility of a targeted music intervention trial in older ED patients and association with clinical outcomes	Quantitative	Prospective pragmatic controlled trial	≥65	<12 h	N.A.
Noel et al.	2019	Risk factors	Emergency department interventions and their effect on deliriums natural course: The Folly May be in the Foley	USA	To identify the modifiable factors of ED care associated with delirium duration in patients admitted to the hospital through the ED	Quantitative	Secondary analysis of a prospective cohort study	≥65	N.A.	N.A.
O’Regan et al.	2018	Occurrence, Risk factors	Predictors of Delirium Development in Older Medical Inpatients: Readily Identifiable Factors at Admission	Ireland	To identify predictors of incident delirium in older medical patients, based on information readily available and easily recordable at the point of hospital admission, and without the need for results of bloodwork, repeated measures (e.g., vital signs), or complex calculations (e.g., illness severity), in an effort to simplify risk stratification in this population	Quantitative	Prospective Observational cohort study	≥70	<36 h	33% prevalent delirium within 36 h (DRS-R98)
Orlandini et al.	2025	Occurrence, Consequences	Factors associated with hospitalization from a geriatric short-stay unit (OBI-GER): a retrospective cohort study	Italy	To characterize the clinical profile of older adults admitted to OBI-GER and identify factors associated with hospitalization within the first 24 h of admission	Quantitative	Observational study	Mean 82, 9	N.A.	Prevalence 22% within 24 h (4AT)
O´Sullivan et al.	2018	Occurrence, Detection	Validation of the 6-Item Cognitive Impairment Test and the 4AT test for combined delirium and dementia screening in older Emergency Department attendees, compared to DSM-IV.	Ireland	To validate 4AT and 6CIT and assess diagnostic accuracy for ED screening for delirium and dementia	Quantitative	Diagnostic accuracy study.	≥70	N.A.	Prevalence 15.2% (DSM-IV)
*Penfold et al.	2023	Occurrence, Consequences	Delirium in hip fracture patients admitted from home during the COVID-19 pandemic is associated with higher mortality, longer total length of stay, need for post-acute inpatient rehabilitation, and readmission to acute services	Scotland	To determine any associations of delirium in patients admitted from home with: 1) mortality at 30 and 180 -days; 2) total length of stay in acute and subacute care settings; 3) need for post-acute inpatient rehabilitation; and 4) readmission to hospital from home within 180 days	Quantitative	Observational study	Mean 82, 9	N.A.	Prevalence in hip fracture: 12,2% (4AT)
Pouw et al.	2023	Occurrence, Detection	Diagnostic accuracy of the Dutch version of the 4AT for delirium detection in a mixed patient population and setting	Netherlands	To evaluate diagnostic accuracy of the Dutch version of the screening tool 4AT for delirium detection in different settings	Quantitative	Prospective observational study	≥65	during ED time	Prevalence 6,1% (DSM-IV)
*Raso et al.	2022	Occurrence, Risk factors	Hospitalizations of older people in an emergency department related to potential medication induced hyperactive delirium: a cross-sectional study	Brazil	To estimate the prevalence of hospitalization of older people with potential medication induced hyperactive delirium in the emergency department (ED) and to identify the risk factors and the medicines frequently associated with the occurrence of the syndrome.	Quantitative	A cross-sectional, retrospective study assessing electronic medical and medication records attached to an ED.	≥60	<24 h	Prevalence 49,2% with hyperactive delirium (record review)
*Rekvad et al.	2021	Occurrence, Detection	Early detection of delirium in acute elderly patients-Systematic use of Confusion Assessment Method	Denmark	To investigate the prevalence of delirium in patients ≥ 65 years old at the Emergency Department Odense University Hospital and to determine whether systematic use of Confusion Assessment Method (CAM) would result in increased identification and diagnosis of delirium.	Quantitative	All caregivers in the Emergency department (ED) at Odense University Hospital received 2 hours of education in delirium and how to use the CAM score. They were asked to systematically perform CAM score in all patients ≥ 65 years at arrival to the ED and at least every 8 hours. During 2 periods of 5 days - one before the caregivers was educated (pre-CAM) and one 4 weeks after CAM scoring was started (during-CAM). A research team interviewed all ≥ 65-year-old patients and their caregivers at the first and second day of admission. In relation to the interviews all patients underwent a cognitive assessment using the CAM by a trained investigator.	≥65	Witihin 24 hours	Prevalence: predata: 24% and postdata 20%. (CAM)
*Rizzi et al.	2015	Occurrence, Risk factors, Consequences	Prognostic Value and Risk Factors of Delirium in Emergency Patients with Decompensated Heart Failure	Spain	To investigate the presence of delirium at admission (prevalent delirium) in ED patients with decompensated heart failure, identify their risk factors, and analyze their impact on clinical outcomes with regard to mortality at 30 days	Quantitative	An observational, prospective, multicentric and cross-sectional study	Mean age 81.7	NA	14.6% delirium in subgroup (bCAM)
Russek et al.	2023	Management	Pharmacological Management of Agitation and Delirium in Older Adults: a Survey of Practices in Canadian Emergency Departments	Canada	To characterize prescribing patterns of medications for agitation by ED physicians in Canadian hospitals	Quantitative	Multisenter survey	N. A.	N.A.	N.A.
Russo et al.	2025	Occurrence, Risk factors	Impact of Living Arrangements on Delirium in Older ED Patients	Italy	To assess how the socio–family demographic status of patients is related to the onset of delirium in a large cohort of older adults aged ≥ 65 years evaluated in the emergency department (ED) using a comprehensive geriatric assessment (CGA)		An observational, prospective, multicentric and cross-sectional study	Mean age 81.7	Not reported	Prevalence: 37.7% (bCAM)
Saito et al.	2025	Prevention	Highdose IV acetaminophen reduces delirium risk in older adults with acute abdominal conditions: a retrospective cohort study	Japan	To evaluate whether the administration of high-dose acetaminophen in older patients diagnosed with acute abdomen in the ED can suppress the onset of delirium during hospitalization	Quantitative	Retrospective cohort study	≥70	N.A.	N.A.
Sanguanwit et al.	2023	Occurrence, Consequences	Thirty-day mortality among patients with acute delirium in the emergency department	Thailand	To evaluate the effect of acute delirium in the ED on 30-day mortality in older patients, including the short-term outcomes ED LOS, hospital LOS, and 30-day ED revisits in older patients with delirium.	Quantitative	A prospective cohort study	≥65	<12 hours	Prevalence 28% (CAM-ICU)
Schnitker et al.	2015	Management	Process Quality Indicators Targeting Cognitive Impairment to Support Quality of Care for Older People with Cognitive Impairment in Emergency Departments	Australia	The aim of this study was to develop an inclusive set of PQIs for the quality of care provided to older people with cognitive impairment presenting to EDs	Other	Quality improvement	≥70	N.A.	N.A.
Schonnop et al.	2022	Detection	Understanding why delirium is often missed in older emergency department patients: a qualitative descriptive study	Canada	The objective of this study was to explore the perceptions of ED physicians and nurses regarding factors contributing to missed delirium in older ED patients	Qualitative	Individual interviews	N. A.	N.A.	N.A.
Seidenfeld et al.	2024	Risk factors	Risk factors and risk stratification approaches for delirium screening: A Geriatric Emergency Department Guidelines 2.0 systematic review	USA	To find risk factors or risk stratification approaches that can be used to identify subsets of older adults who may benefit from targeted ED delirium screening	Review	Systematic review	≥65	N.A.	N.A.
*Sethi et al.	2025	Occurrence	Prevalence of Psychiatric Morbidity Among the Elderly Patients Presenting to Emergency Trauma Setting: An Exploratory Study	India	To evaluate the prevalence of psychiatric morbidity among elderly patients presenting to the Emergency trauma setting	Quantitative	Cross-sectional study, with a convenient sampling technique.	≥60	24 h	Prevalence: 24% (DSM-V)
Silva et al.	2022 b	Occurrence, Risk factors	Association between emergency department modifiable risk factors and subsequent delirium among hospitalized older adults	USA	To evaluate the association between potential ED-based modifiable risk factors and subsequent development of delirium among hospitalized older adults	Quantitative	Observational study	≥75	N.A.	Incidence 7% (modified bCAM) within 48 h
*Silva et al.	2022a	Occurrence, Risk factors	REcognizing DElirium in geriatric Emergency Medicine: The REDEEM Risk Stratification Score	USA	To derive a risk score that uses variables available early during the ED encounter to identify high-risk geriatric patients who may benefit from delirium screening	Quantitative	Observational study	≥75	N.A.	Prevalence 11.1% (DTS and bCAM)
*Silva et al.	2021	Risk factors	Risk Factors for Delirium in Older Adults in the Emergency Department: A Systematic Review and Meta-Analysis	N.A	To identify risk factors for delirium in geriatric ED and find modifiable risk factors for delirium development	Quantitative	Systematic review and meta-analysis including papers	≥60	N.A.	N.A.
Snapp et al.	2024	Prevention	Reduction of Postoperative Delirium and Opioid Use in Hip Fracture Patients Through Utilization of Emergency Department Physician Administered Regional Nerve Blocks	USA	To evaluate the utilization of ED physicians to perform fascia iliaca nerve blocks on hip fracture patients to decrease the incidence of delirium by decreasing usage of opioid medication	Quantitative	Quality improvement	93%>60	N.A.	N.A.
Soler-Sanchis et al.	2024	Occurrence, Detection	The 4AT scale for rapid detection of delirium in emergency department triage	Spain	To assess the diagnostic accuracy and time impact of the 4AT scale in emergency department triage	Quantitative	A Prospective diagnostic accuracy study	≥65	NA	Prevalence 41.4% (ICD-10)
Soler-Sanchis et al.	2022	Risk factors	Challenges in the Detection of Clinically Useful Biomarkers for the Diagnosis of Delirium in Older People in the Emergency Department—A Case–Control Study	Spain	To determine whether there are clinically useful biomarkers recorded in the ED for application to older people with delirium	Quantitative	Case-control	≥65	N.A.	N.A.
Soler-Sanchis et al.	2023 **a**	Consequences	Clinical Risk Group as a predictor of mortality in delirious older adults in the emergency department	Spain	To assess whether chronicity, as assessed by Clinical Risk Groups (CRG), is an independent predictor of mortality in older adults with delirium seen in the ED	Quantitative	Prospective study with 18-month follow-up	≥65	NA	N.A.
Soler-Sanchis et al.	2023**b**	Risk factors	Identification through the Manchester Triage System of the older population at risk of delirium: A case– control study	Spain	To identify the flow charts and discriminators of the Manchester Triage System that are most likely to identify the onset of delirium in older people	Quantitative	A retro-spective, case and non-matched control study	≥65	NA	N.A.
Tan et al.	2022	Management	Computerized tool and interdisciplinary care for older patients with delirium in the emergency department: a novel model in Taiwan	Taiwan	A computerized tool and interdisciplinary care were implemented to develop a novel model for older patients with delirium in the ED	Quantitative	Implementation	≥65	NA	N.A.
Thao et al.	2025	Occurrence, Detection	Validation of a chart review method for identifying delirium in the emergency department	not reported	To validate the Chart-based Delirium Identification Instrument (CHART-DEL) which was developed in the inpatient setting, for identifying delirium in the emergency department (ED).	Quantitative	Retrospective observational study	≥75	not reported	Prevalence: 4.4% (DTS/bCAM)
Tong et al.	2016	Detection	A Serious Game for Clinical Assessment of Cognitive Status: Validation Study	Canada	To demonstrate the feasibility of a game-based cognitive assessment delivered on tablet technology to a clinical sample and to conduct preliminary validation against standard mental status tools commonly used in elderly populations	Quantitative	Prospective observation clinical study	≥70	≥4hpted	N.A.
Traynor et al.	2016	Occurrence, Detection	Is delirium being detected in emergency?	Australia	To report on the use of Delirium Care Pathways to screen for and recognise delirium by Aged Care Services in Emergency Teams (ASETs) at five metropolitan hospitals in New South Wales, Australia	Quantitative	Brief report: Audit of medical records	≥65	N.A.	Prevalence 14% (record audit- symptoms of delirium)
Van de Meeberg et al.	2016	Detection	Improved detection of delirium, implementation and validation of the CAM-ICU in elderly Emergency Department patients	Netherlands	To evaluate the effect of routine use of the Confusion Assessment Method for the Intensive Care Unit (CAM-ICU) on the diagnosis rate of delirium in elderly Emergency Department (ED) patients and the validity of the CAM-ICU in the ED setting	Quantitative	Prospective observational study	≥70	N.A.	Prevalence: Pre 3%, and after 10% (DSM-IV)
*van Loveren et al.	2021	Risk factors	Increased Emergency Department Hallway Length of Stay is Associated with Development of Delirium	not reported	To determine 1) the association between time spent in the emergency department (ED) hallway and the development of delirium and 2) the hospital location of delirium development	Quantitative	Retrospective cohort study	Median 71	N.A.	N.A
Vardy et al.	2020	Detection	Use of a digital delirium pathway and quality improvement to improve delirium detection in the emergency department and outcomes in an acute hospital	UK	To screen 65% of those admissions aged 65 years and over from the A&E department for delirium by March 2018	Other	Quality improvement	≥65	N.A.	N.A
Voyer et al.	2017	Detection	RADAR: A rapid detection tool for signs of delirium (6th- vital sign) in emergency departments	Canada	To validate the RADAR for the detection of the 6th vital sign in the ED	Quantitative	Validation study. Part of a larger multicentre project, the INDEED study	≥65	≥8 hours	N.A
Yadav et al.	2019	Occurrence, Detection	Serial Ottawa 3DY assessments to detect delirium in older emergency department community dwellers	Canada	To determine the sensitivity and specificity of serial O3DY assessments to detect delirium in older ED patients	Quantitative	Prospective observational multicenter cohort study (4 EDs)	≥65	≥8houers	Prevalence 10% (CAM) (in ED)
Zucchelli et al.	2021	Risk factors	Development and validation of a delirium risk assessment tool in older patients admitted to the Emergency Department Observation Unit	Italy	To develop and validate a delirium risk assessment tool for older persons admitted to the ED Observation Unit (OU)	Quantitative	Retrospective cohort study	≥65	N.A.	N.A.
Émond et al.	2017	Occurrence, Risk factors, Consequences	Emergency Department Stay Associated Delirium in Older Patients	Canada	To assess the incidence and impacts of ED stay associated delirium	Quantitative	Record review	≥65	≥12 hours	ED related delirium: Incidence of 18% (Chart based CAM)
´Émond et al.	2018	Occurrence, Risk factors	Incidence of delirium in the Canadian emergency department and its consequences on hospital length of stay: a prospective observational multicentre cohort study	Canada	To determine the incidence of delirium and describe its impacts on hospital length of stay among non-delirious community-dwelling older adults with an 8-hour exposure to the emergency department (ED) environment.	Quantitative	A prospective observational multicentre cohort study (March–July 2015).	≥65	≥8 hours	The mean incidence of ED-related delirium was 12.1% (*n* = 41) (CAM)

In publications including a wider age group, we examined the publication more thoroughly and included the paper if the mean or median age was > 60 years or if it was apparent that the publication aimed at initiatives and care for the older patient group.

In publications that included a broader perspective than delirium in the elderly and or other settings than the ED, we studied the papers in depth and included publications when it was possible to extract data regarding delirium in the ED among older patients. For review papers on delirium where data relevant to older patients in the ED could not be extracted, we screened all included papers to determine whether individual studies could be incorporated into this scoping review.

## Data analysis

All authors were involved in the data analysis in several group meetings. The content of the publications was discussed and categorised using a simple qualitative content analysis [14]. Several publications covered multiple aspects concerning older patients with delirium in the ED, thus the included publication could be classified under more than one category.

## Results

**Fig. 1:**
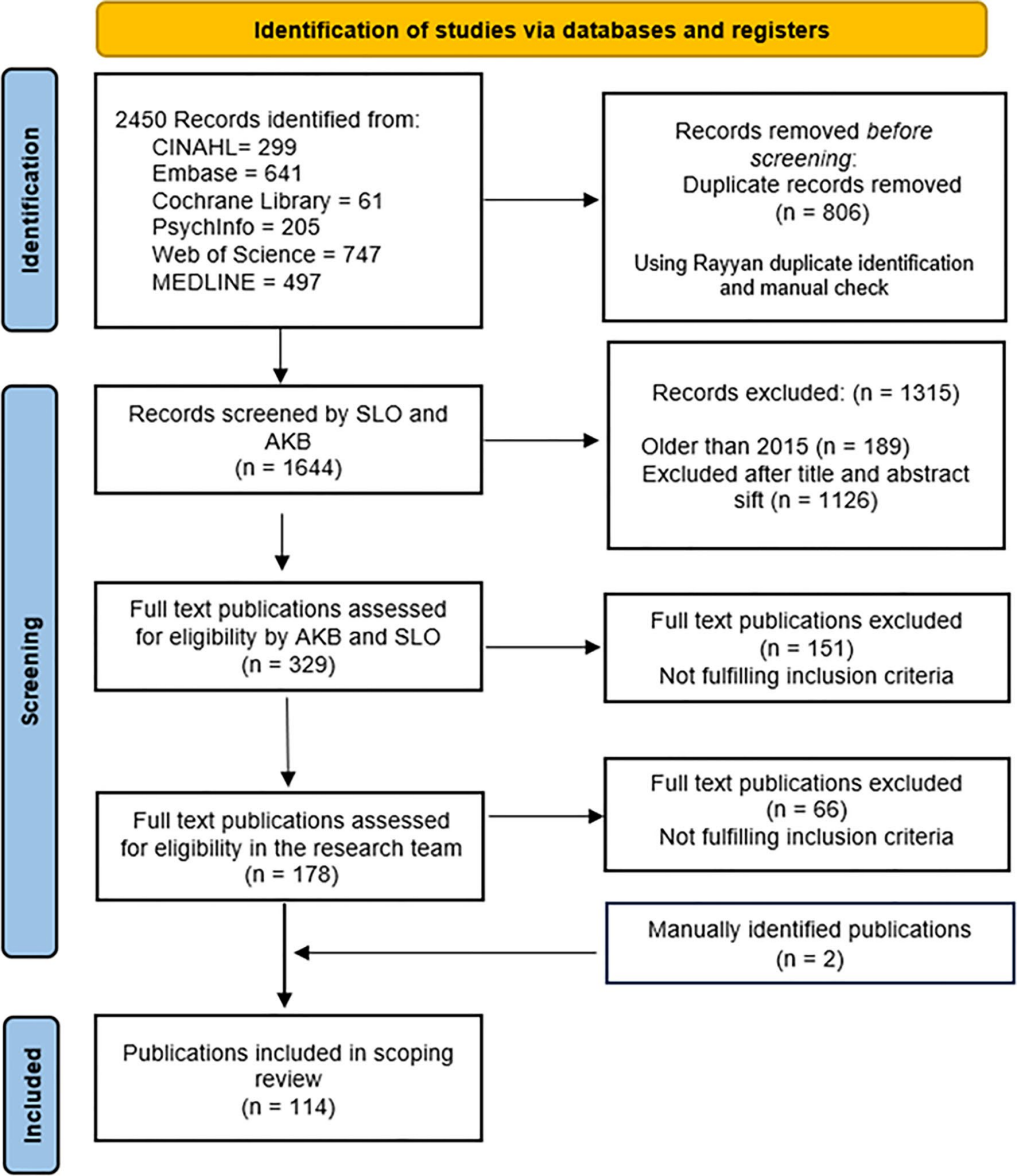
PRISMA flow chart of the data inclusion process

We included a total of 114 publications (Fig. 1, Table 1), including 19 publications where only the relevant information regarding ED were included.

## Overall description of the publications

**Fig. 2:**
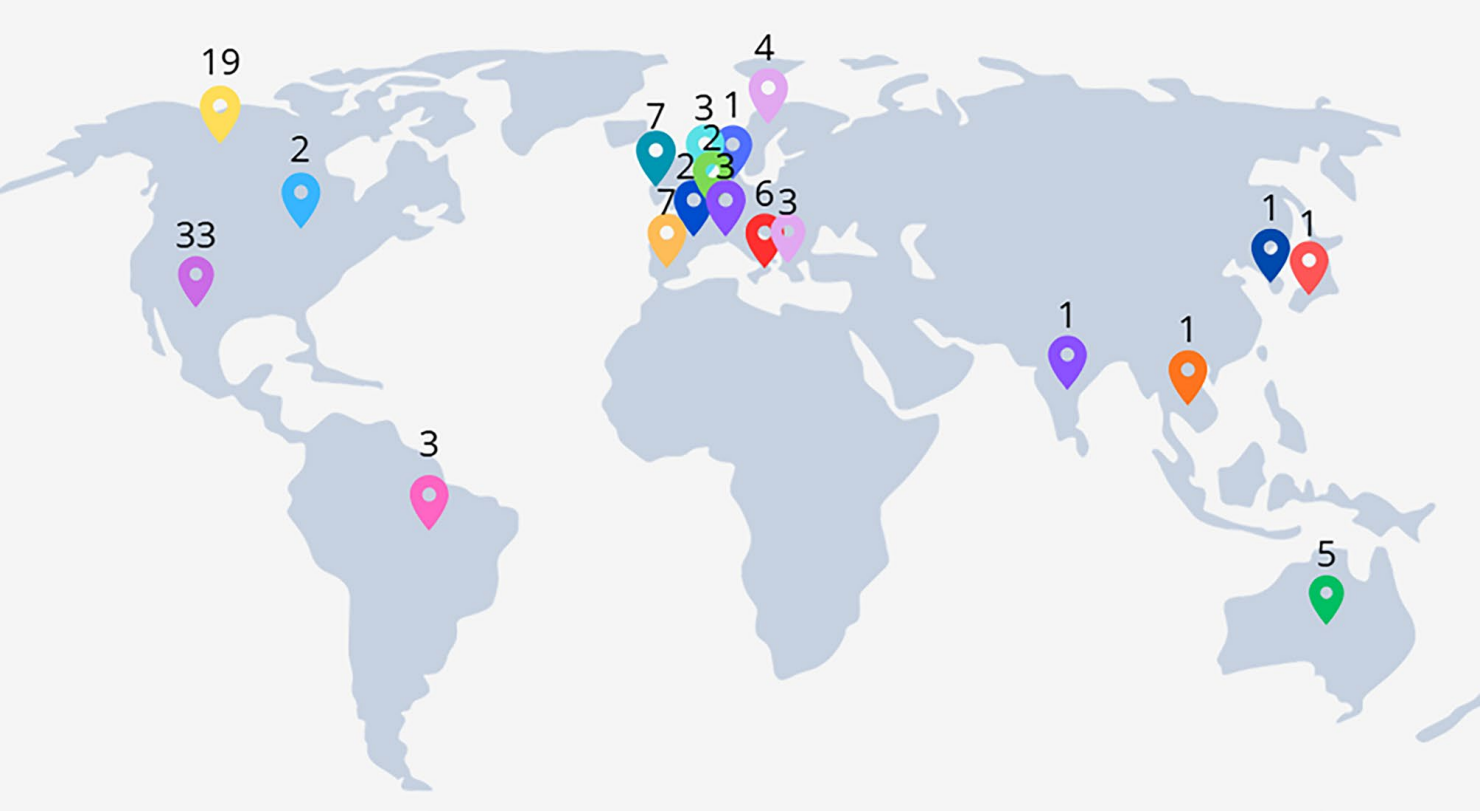
Geographic distribution of studies: country and number of publications: Australia 5, Brazil, 3 Canada, 19, Denmark 1, France 2, Germany 2, India 1, Ireland 4, Italy 6, Japan 1, Korea 1, Netherlands 3, Norway 4, Scotland 1, Spain 7, Switzerland 3, Taiwan 1, Thailand 1, Turkey 3, USA 33, USA and Canada: 2, UK: 2. (not reported: 2, several countries in reviews: 7)

The publications represented different regions from all continents besides Africa, with a majority originating from North America (*N* = 54) and Europe (*N* = 35) (Fig. 2, Table 1).

**Fig. 3:**
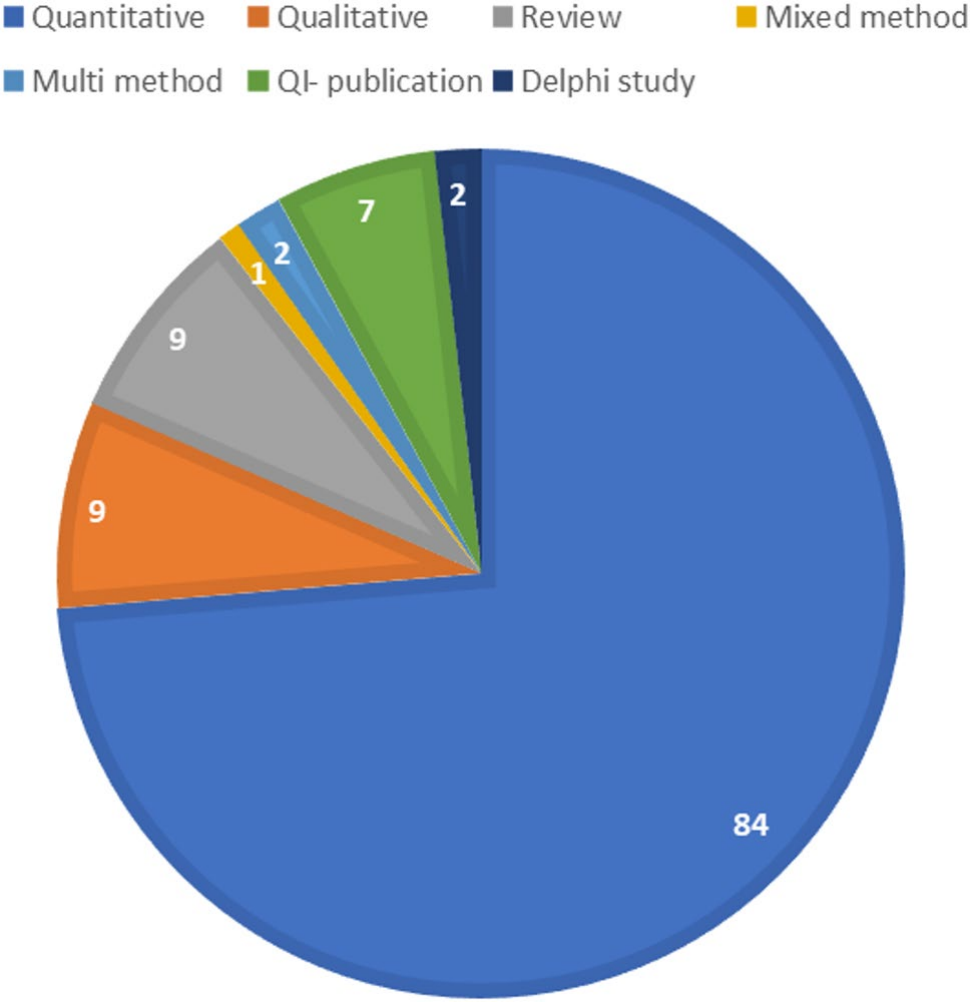
Distribution of main methodologies

Most of the studies applied a quantitative method 74% (*n* = 84). Qualitative original publications, review papers and QI-projects constituted 7.0% (*n* = 9) 7.0% (*n* = 9) and 6,7% (*n* = 7) respectively (Fig. 3). Furthermore, most quantitative publications included patients ≥ 65 years of age (*n* = 58), while 18, (24,6%) chose an older age group (≥ 70 or ≥ 75), and a few (*n* = 4) chose to have ha lower cut off ≥ 60.

### Categorisation of the included publications

**Fig. 4:**
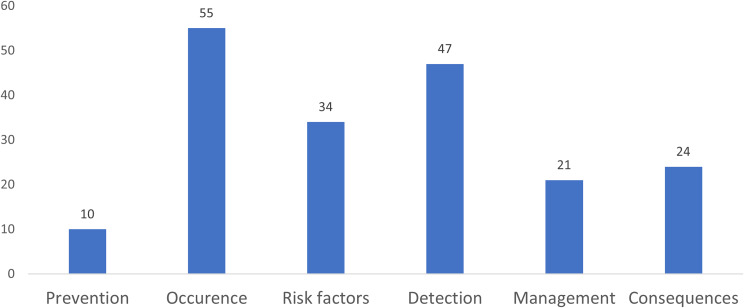
Distribution of main categories (numbers of publications)

Through a simple content analysis [14], we categorised all publications into six main categories: *Prevention, Occurrence, Risk factors, Detection, Management and Consequences.* Occurrence and Detection were the two largest categories including *n* = 55 and *n* = 47 of the publications respectively, while fewest publications (*n* = 10) were categorised as Prevention (Fig. 4). The nine included review publications and categories are presented in Table 2.

**Table 2:** Overview over review

Author	Type of review	Main categories
Ehrlich et al. 2024	Narrative review	Prevention, Management
Seidenfeld et al. 2024	Systematic review	Risk factors
Carpenter et al. 2024	Systematic review and meta-analysis	Detection
Bonfichi et al. 2023	Narrative review	Prevention, Detection, Risk factors, Management
Filiatreault et al. 2023	Umbrella review of clinical practice guidelines	Prevention, Detection, Management
e Silva et al. 2021	Systematic review and meta-analysis	Risk factors
Lee S. et al. 2023	Systematic review and meta-analysis	Management,
Lee S. et al. 2022	Systematic review	Prevention, Management
Badawy & Shaban 2025	Systematic review	Management

The 105 original research publications were further subcategorised to provide more detailed information about their findings (Table 3).

**Table 3:** Table of categories and subcategories of original research publications

Categories	Subcategories	Publications
Prevention	Physical environment initiatives	Ní Chróinín et al. 2025, Keene et al. 2023
	Fundamental patient care	Chary et al. 2022b, Kennedy et al. 2021
	Medical interventions	Snapp et al. 2024, Saito et al. 2025
Occurrence	Prevalence	Hsieh et al. 2015, Han et al. 2015, Dyer at al. 2016, Han et al. 2016, Traynor et al. 2016, Mota et al. 2016, Han et al. 2017a, Grossman et al.2017, Baten et al. 2018, Marra et al. 2018, Gagné et al.2018, Haseman et al. 2018, Bédard et al. 2019, Brich et al. 2019, Choutko-Joaquim et al. 2019, Evensen et al. 2019, Lucke et al. 2019, Lee S. et al. 2018, Yadav et al. 2019, Mailhot et al. 2020, Rekvad et al. 2021, Lee J. et al. 2022, Lee S. et al. 2022b, e Silva et al. 2022a, Raso et al. 2022, Arneson et al. 2023, Pouw et al. 2023, Sanguanwit et al. 2023, Soler- Sanshis et al. 2024, Morales-Puerto et al. 2024, Marcomini et al. 2024, Russo et al. 2025, Thao et al. 2025, *Within 48 hours: Nguyen et al. 2018, O´Sullivan et al. 2018, O´Regan et al. 2018
	Prevalence in specific diagnostic subgroups	Rizzi et al. 2015, Elsayem 2016, Elsayem et al. 2017, Aslaner et al. 2017, Penfold et al. 2023, Demirtakan et al. 2023, Sethi et al.2025, Goll et al. 2025, Orlandini et al. 2025 (within 48 h)
	Incidence in ED	Keene et al. 2023 (*subgroup), Miró et al. 2024
	ED- related incidence (within 48 h of ward arrival)	Émond et al. 2017, Émond et al. 2018, Evensen et al. 2018, Giroux et al.2018, Gagné et al. 2018, Boucher et al. 2019, Daoust et al. 2020, Béland et al. 2021, Giroux et al. 2021, Silva et al. 2022b
Risk factors	Patient related factors	Rizzi et al. 2015, Mota et al. 2016, Nguyen et al. 2017, Giroux et al.2018, Cirbus et al. 2019, Choutko-Joaquim et al. 2019, McNeil et al. 2019, Noel et al. 2019, Béland et al. 2021, Daoust et al. 2020, Miró et al. 2024, Zucchelli et al. 2021, Marcomini et al.2024, Joseph et al. 2024, Lafuente-Lafuente 2025, Russo et al. 2025, Goll et al. 2025
	Pharmacological factors	Nguyen et al. 2017, Han et al. 2019, Noel et al. 2019, Daoust et al. 2020, Giroux et al. 2021, Raso et al. 2022, Lee S. et al. 2022b, Silva et al. 2022b, Miró et al. 2024, Marcomini et al.2024
	Patient flow factors	Émond et al. 2017, Émond et al. 2018, Evensen et al. 2018, Béland et al. 2021, Giroux et al. 2021, van Loveren et al. 2021, Silva et al. 2022b, Elder et al. 2023, Miró et al. 2024, Joseph et al. 2024
	Diagnostics, treatment and care	Noel et al. 2019, Béland et al. 2020, Silva et al. 2022b, Marcomini et al. 2024
	Risk stratification	O`Regan et al. 2018, Zucchelli et al. 2021, Silva et al. 2022a, Soler-Sanschis et al. 2022, Soler-Sanschis et al.2023b, Lafuente-Lafuente et al.2025,
Detection	Screening tool diagnostic performance	**Versus DSM-IV:** Han et al. 2015 (RASS), Han et al. 2016 (bCAM), Grossman et al. 2017 (mRASS), Han et al. 2018 (Single question), Marra et al. 2018 (MOTYB/MOTYB-6 and LUNCH), Hasemann et al. 2018 (mCAM ED tool) **Versus ICD-10:** Soler-Sanchis et al. 2024 (4AT) **Versus CAM:** Tong et al. 2016 (Game based assessment), MacLullich et al. 2019 (4AT), Yadav et al. 2020 (O3DY), Versus *Composite reference: Clinical impression, CAM ICU and DRS-R98 > 18: Lee et al. 2018 (BSEEG) **Comparing screening tools**: Lucke et al. 2019 (CAM-ICU vs 6CIT)
	Screening tool validation	**Versus DSM IV:** van de Meeberg et al. 2017 (CAM ICU), Baten et al. 2018 (bCAM), O´Sullivan et al. 2018 (4AT), Brich et al. 2019 (nuDESC),Pouw et al. 2023 (Dutch 4AT) **Versus CAM**: Voyer et al. 2017 (6^th^ vital sign), Gagné et al. 2018 (4AT in French), Bédard et al. 2019 (O3DY in French), Mailhot et al. 2020 (FAM-CAM) **Versus DRS-R-98**: Morales-Puerto 2024 (4AT in Spanish) **Versus DTS and bCAM**: Thao et al. 2025 (CHART-DEL)
	Increasing delirium detection	Arendts et al. 2017 van de Meeberg et al. 2017, Hullick et al. 2018, Vardy et al. 2020, Buterakos and Keiser 2021, Rekvad et al. 2021, Minion et al. 2021, Martin et al. 2022
	Detection of delirium in clinical practice	Elsayem et al. 2016, Traynor et al. 2016, LaMantia et al. 2017, MacLullich et al. 2019, Kennedy et al. 2021, Eagles et al. 2022, Lee J. et al. 2022, Schonnop et al. 2022, Chary et al. 2022, Chary et al. 2023, Fossen and Bergland 2023, Chary et al. 2024, Chary et al. 2025, Mailhot et al. 2021
Management	Factors in ED- environment	LaMantia et al. 2017, Ní Chróinín et al. 2025
	Dedicated personnel	Hullick et al. 2018, Buterakos & Keiser 2021
	Multimodal approaches for patient care	Buterakos & Keiser, Kennedy et al. 2021, Mailhot et al. 2021, Tan et al. 2022, Asiss et al. 2022, Filiatreault et al.2024
	HCPs perceptions on management	LaMantia et al. 2017, Chary et al. 2021, Chary et al. 2025
	Pharmacological management	LaMantia et al. 2017, Kennedy et al. 2021, Russek et al. 2023,
	Identifying and treating underlying conditions	Dyer et al. 2016, Kennedy et al.2021, Butcher et al. 2022
	Quality performance	Schnitker et al. 2015, Filiatreault et al.2024
Consequences	Worsening clinical condition	Hsieh et al. 2015, Han et al. 2017b, Boucher et al. 2019, Cirbus et al. 2019, Giroux et al. 2021
	Increased need of healthcare	Hsieh et al. 2015, Émond et al. 2017, Émond et al. 2018, Elsayem et al.2017, Ma et al. 2018, Boucher et al. 2019, Mailhot et al. 2020, Penfold et al. 2023, Orlandini et al. 2025
	Mortality	Rizzi et al. 2015, Hsieh et al. 2015, Elsayem et al.2017, Han et al. 2017**a**, Israni et al. 2018, Lee et al. 2022b, Mailhot et al. 2020, Penfold et al. 2023, Miró et al. 2024, Arneson et al. 2023, Demirtakan et al. 2023, Sanguanwit et al. 2023, Russo et al. 2025
	Mortality predictor	Soler-Sanchis 2023a, Dogan et al. 2024, Cereda et al. 2025

### Prevention

Ten publications presented findings regarding preventing patients from developing delirium in the ED or in relation to the ED stay [16–25] (Tables 1, 2 and 3, Supplementary file B). This includes two narrative reviews [18, 19], one systematic review [21] and one umbrella review over clinical guidelines [20]. The six original research publications included in this category were further subcategorised into three subcategories. *Physical environment initiatives* included publications evaluating factors regarding the use of light and or music therapy in the ED setting in relations to delirium [16, 17]. The qualitative study included in the subcategory *Fundamental patient care* [22] described how ED nurses on ad- hoc basis focused on fundamental care such as sensory devices, food drink and toilet protocol to care for older ED- patients to prevent delirium. This focus is consistent with The ED DEL change-package and tool kit presented by Kennedy et al. [23], highlighting the importance of providing fundamental care for patients at risk of delirium, such as nutrition, hydration, mobility, sensory devices, nocturnal rhythm, and provide orienting communication. In the subcategory *Medical interventions,* two publications studied interventions to reduce pain in older ED patients. Snapp et al. [25] found an increase in usage of regional nerve blocks in older hip fracture patients reduced postoperative delirium and use of opioids. Saito et al. [24] demonstrated that high dose acetaminophen intravenously was associated with a reduction in delirium risk in older patients with acute abdominal conditions.

### Occurrence

A total of 55 publications presented data of occurrence of delirium in the ED (Tables 1, 2, 3 and Supplementary file B), including *Prevalence* of delirium in the ED [26–67], within 48 h of arrival [45, 68, 69] *Incidence of ED delirium* [16, 49] or *Incidence of ED- related delirium* -*occurring within 48 h of ward arrival* [70–78]. A variety of tools or reference standards were used to assess occurrence of delirium (Tables 1, 4). The Confusion Assessment Method (CAM) was the most frequently used method for assessing occurrence (*n* = 16), followed by the 4 A´s Test (Alertness, Abbreviated mental test, Attention, Acute change (4AT)) (*n* = 11) (Table 4). The reported prevalence of delirium in the included publications in the ED varied between 1,3% in a study using CAM- ICU [44], to 49,2% in record review using DSM-V [53] (Table 1). Incident delirium within 48 hours of ward arrival varied between 1.6% [49] to 18% [72].

**Table 4:** Overview over screening tools used in the included original research publications

Screening tool	Publications
bCAM	Rizzi et al. 2025, Han et al. 2017b, Aslaner et al. Baten et al. 2018, Minion et al. 2021, Buterakos and keiser 2021, Russo et al. 2025, Thao et al. 2025, Arneson et al. 2023, e Silva et al. 2021
CAM	Soler-Sanschis et al. 2023a, Keene et al. 2023, Shenkin et al. 2019, Gagné et al. 2018, Giroux et al. 2018, Giroux et al. 2021, Choutko-Joaqim et al. 2019, Elsayem et al. 2017, Elsayem et al. 2017, Sieben-daMota 2016, Bedard et al. 2019, Shenkin et al. 2019, Mailhot et al. 2020, Daoust et al. 2020, Béland et al. 2021, van Louvren et al. 2021, Rekvad et al. 2021
CAM ICU	Hsieh et al. 2015, Dyer et al. 2016, Elsayem et al. 2017, Han et al. 2017a, Lucke et al. 2019, Van de Meerberg et al. 2017, Lee S et al. 2018, Sanguanwit et al. 2023
FamCAM	Mailhot et al. 2020
Modified bCAM	Han et al. 2016, e Silva et al. 2022b.
NuDESC	Brich et al. 2019
Nurse screen	Arendts et al. 2017
4AT	Gagné et al. 2018, O´Sullivan et al. 2018, MacLullich et al.2019, Vardy et al. 2020, Martin et al. 2022, Demirtakan et al. 2023, Pouw et al. 2023, Penfold et al. 2023, Marcomini et al. 2024, Soler-Sanschis et al. 2024, Orlandini et al. 2025.
DTS	Thao et al. 2015, Arneson et al. 2023, e silva et al. 2021
BS-EEG	Lee S.et al. 2018
RADAR (6^th^ vital sign)	Voyer et al. 2017
Serial OTTAWA 3DY	Yadav et al. 2019
mCAM ED tool	Haseman et al. 2018
Chart based CAM tool	Émond et al. 2017
mRASS	Grossmann et al. 2017
Chart DEL	Thao et al. 2025
6-CIT	Lucke et al. 2019
MOTYB-12/ MOTYB-6	Marra et al. 2018
LUNCH	Marra et al. 2018
Single question evaluation	Han et al. 2018
DRS-R98	O`Regan et al. 2018, Morales-Puerto et al. 2024, Lee S. et al. 2018
RASS	Han et al. 2015
Delirium index	Bocher et al. 2019
DOSS	Lee S et al. 2022b
MDAS	Elsayem et al. 2017

bCAM= Brief Confusion Assessment method, CAM= Confusion Assessment method, CAM ICU= Confusion Assessment method for the intensive care unit, FamCAM= Family Confusion Assessment method, Modified bCAM= Modified Brief Confusion Assessment method, NuDESC= Nursing Delirium Screening Scale, Nurse screen= ED Nurse Delirium Screening form, 4AT= the 4 A´s Test (Alertnes, Abbreviated mental test, Attention, Acute change), DTS= Delirium Triage Screen, BS- EEG= Bispectral EEG, RADAR= Recognizing Acute Delirium As part of your Routine (the 6th vital sign), OTTAWA 3DY= what Day, what Date, spell worlD, Year, mCAM ED tool= modified Confusion Assessment Method for the Emergency Department, Chart based CAM tool= Method for using CAM in a record review , mRASS= The modified Richmond Agitation Sedation Scale, Chart DEL= Chart- based Delirium Identification Instrument, 6-CIT= 6-Item Cognitive Impairment Test, MOTYB-12/ MOTYB-6= Reciting the months of year/6 months backwards,  LUNCH=Spelling the word LUNCH backwards, Single question evaluation= ‘Have you had any difficulty thinking clearly today?’, DRS-R98= Revised Delirium rating Scale, RASS= Richmond Agitation Sedation Scale, Delirium index= Delirium severity tool, DOSS= Delirium Observation Screening Scale, MDAS= Memorial delirium assessment scale

### Risk factors

Risk factors for ED- and ED related delirium were studied in 34 publications [18, 31, 37, 47, 49, 53, 55, 58, 59, 64, 66, 68–76, 78–91] (Tables 1, 2, 3 Supplementary file B) including three review- papers [18, 86, 87]. The original studies were subcategorized into 5 subcategories (Table 3).

The subcategory *Patient factors* included publications reporting on risk factors like advanced age [74, 78, 89], cognitive impairment/dementia [47, 55, 68, 69, 74, 76, 79, 82, 89], lower educational level [58], frailty [31, 37, 64, 75], reduced functional ability [55, 69, 74] and sensory loss, such as hearing impairment [69, 89], metabolic- electrolyte and fluid disturbance [47, 79, 83], systemic inflammation/infections and organ dysfunction [68, 79, 83, 84] and pain [78].

The *Pharmacological factors* subcategory encompassed publications evaluating the risk of delirium related to potentially inappropriate medications (PIMs), such as opioids, benzodiazepine, anticholinergic drugs [49, 53, 66, 70, 72, 78, 85] and psychotropic drugs [47, 81]. In addition, a study validating the Delirium Drug Scale [68].

Further, *ED- patient-flow factors* covered publications studying ED-length of stay (LOS) [49, 71–74, 76, 80, 82, 88], ED- hallway LOS and [80, 88] ED-boarding time in [82] relations to development of delirium. While most studies found an association between their studied patient flow factor and delirium, Evensen et al. [73] and Elder et al. [80] did not.

The subcategory of *ED diagnostics, treatment and -care* included studies finding foley catheter placement [70, 85] and immobilization [76] in the ED associated with delirium. Lastly, *Risk assessment,* encompassed publications focusing on identifying predictors of delirium development [59, 89, 90, 92], including the development of a delirium risk assessment tool [89] and the use of a triage system to identify older patients at increased risk [59].

### Detection

Nearly half of the publications (*n* = 50) focused on aspects of delirium detection in older ED patients [18, 20, 22, 23, 28–30, 34, 36, 38–40, 42, 44–46, 48, 50, 51, 54, 60–63, 65, 90, 92–115] (Tables 1, 3). Of these, three were review studies [18, 20, 96] (Table 2). Five subcategories encompassed the focus of the 47 original studies (Table 3). Several different screening tools were applied in these publications (Table 4).

The first subcategory contained a variety of S*creening tools- and their diagnostic performance* in the ED, of which seven compared the studied screening tool to a standard reference using Diagnostic and Statistical Manual of Mental Disorders, Fourth Edition DSM-IV [38–40, 42, 48, 102] or ICD-10 [60]. Furthermore, 12 studies reported on *Screening tool validation* including the validation of 4AT screening tools in Dutch [51], Spanish [50], and French [36] and the OTTAWA 3DY (O3DY) in French [29].

Moreover, *Increasing delirium detection* in the ED was the focus in eight studies, describing initiatives to improve delirium detection in the ED [54, 94, 95, 103, 106, 110, 113, 114]. The subcategory *Detecting delirium in clinical practice* included three quantitative publications studying to what degree Health Care Personnel (HCP) managed to identify patients with delirium in the ED [34, 62, 65], all reporting quite low detection rates. Traynor et al. [62] reported that of 31 patients with symptoms of delirium, only four had a delirium diagnosis documented in their medical records. Furthermore, the eight qualitative studies included in this subcategory [97–101, 104, 111, 116], explored facilitators and barriers for delirium detection through interviews with HCPs. Barriers included the busy nature of the ED [104], need for increased knowledge about delirium [100, 104, 111] and delirium screening as a deprioritized activity [100, 111]. The recent study by Chary et al. [98] highlighted how ED nurses believed that implementation of delirium screening was feasible, but hinged on adequate resources, staffing, leadership support, and time to perform the screening. Finally, this subcategory included two interventions/tool kit development publications [23, 105] aiming to support HCPs in delirium detection and management in the ED.

### Management

21 publications addressing delirium management in the ED were identified [17–21, 23, 33, 83, 95, 98, 103–105, 108, 117–124] (Tables 1, 2, 3). These included six reviews [18–21, 117, 121] (Table 2), four quality improvement studies [23, 95, 105, 123] and one Delphi study [120]. The 14 original research articles were categorized into six subcategories (Table 3).

The subcategory *Factors in ED- environment* included a qualitative study by LaMantia et al. [104] where the participants (medical emergency service providers, nurses and physicians) described the busy and uncontrollable environment of the ED as the greatest challenge when managing delirious patients. Moreover, the subcategory encompassed a pilot study by Ní Chróinín et al. [17] testing the feasibility of music intervention in the ED for better management of delirious patients. No between‑group differences were observed for improved or resolved delirium, pain scores, or agitation/sedation scores, although the intervention itself was deemed feasible and suitable for further large‑scale investigation.

*Dedicated personnel* contained two studies. Hullick et al. [103] evaluated whether the use of Older Persons Technical Assistants (OPTAs) would change screening rates and patient care and found significant improvements in both the rates of documented screenings and the supportive care. Buterakos & Keiser [95] investigated whether a geriatric resource team providing consultation and follow‑up for ED patients who screened positive for delirium could reduce hospital LOS or affect discharge disposition. Their results indicate that a structured delirium screening protocol with a dedicated team applying evidence‑based measures may lessen the negative impact of delirium on both LOS and discharge outcomes.

The subcategory M*ultimodal approaches for patient care* comprised six publications. A study by Assis et al. [119] evaluated the feasibility of involving patients next of kin for better screening and care for older ED patients. They found that having relatives implementing preventive interventions such as food assistance, fluid replacement and early mobilization was a viable strategy to improve care for older ED patients in risk of developing delirium. Tan et al. [124] evaluated an interdisciplinary care model using a computerized tool, education and a continuous care plan and found promising results. Multimodal approaches were also recommended in the tool kit presented by Kennedy et al. [23] the SCREENED-ED intervention by Mailhot et al. [105] and the bCAM protocol described by Buterakos & Keiser [95]. Filiatreault et al. [120] observed that a multicomponent management plan initiated while the patient was still in the ED was important for the quality of delirium management during the hospital stay.

Further, *HCP perceptions on management* included two interview-based studies exploring HCPs perceptions on managing patients with delirium in the ED [98, 104, 108]. HCP acknowledged the non-delirium friendly environment in EDs and requested education about the condition, easy screening tools and knowledge of managing agitated and delirious patients. In the study by Kennedy et al. [23] the interviewees underlined the importance of leadership involvement when implementing initiatives to better the management of geriatric patients in the ED.

*Pharmacological management* included a study by Lamantia et al. [104] where the physicians identified medications as an important option when managing patients with delirium, while the participating nurses described uncertainty when the physicians prescribed multiple medications with overlapping indications. Russek et al. [122] studied the medications used to manage agitation in the ED and observed a more favourable use of medications when standardized order sets were used. Kennedy et al.`s [23] ED-DEL change package and toolkit underscored a cautious use of pharmacological approaches and to involve a pharmacist in the medical management of delirious patients.

In the Subcategory *Identifying underlying conditions,* three publications where included. The previously mentioned study by Kennedy et al. [23] emphasized the importance of evaluating and treating underlying conditions of delirium. A study that assessed the value of a computed tomography (CT) scan of the head in delirious patients suggested minimal utility [118], while an assessment of informant history for patients with delirium concluded that informant history is underutilised and thus recommended increased use [33].

In the *Quality performance* subcategory, Schnitker et al. [123] and Filiatrault et al. [120] describe the development of ED quality performance measures for delirium care. Schnitker et al. [123] proposed three process indicators—delirium screening, risk assessment and assessment of possible causes—to support care for cognitively impaired patients. Filiatrault et al. [120] reached consensus on nine quality statements and 23 performance measures covering screening, diagnosis, risk reduction and management. The management‑related statements included systematic assessment, a multicomponent care plan, appropriate medication use and provision of information to patients and families.

### Consequences

This category included 24 publications [26, 32, 41, 43, 46, 49, 52, 55, 56, 64, 66, 67, 71, 72, 74, 77, 79, 92, 125–130] presented in four subcategories (Tables 1, 3).

*Worsening clinical condition* included six publications [43, 74, 77, 79, 125] studying consequences for the patients after an episode of delirium. Findings included that delirium was associated with clinical deterioration during [43] and after hospitalisation [43, 77] and worsening cognition after six months follow up [74, 125].

Further on, publications gathered in the subcategory *Increased need of healthcare services* [43, 46, 52, 67, 71, 72, 77, 126, 127] studied for instance, the association between ED delirium and the need for hospitalization and hospital LOS. The study by Émond et al. [71] reported ED delirium to be associated with increased mean hospital LOS, Cirbus et al. [79] observed a rise in ED visits over the subsequent 6 months, while Elsayem et al. [126] and Hsieh et al. [43] noted higher rates of Intensive care unit (ICU) admissions.

Moreover, the subcategory *Mortality* contained 12 publications studying the association between ED-delirium and mortality [26, 32, 41, 43, 46, 49, 52, 55, 56, 64, 66, 126, 128]. While Penfold et al. [52] found delirium to be associated with mortality (within 180 days), Sanguanwit et al. [56] did not (within 30 days). Finally, three publications studied *Mortality predictors* in delirium patients [92, 129, 130], including the study by Soler-Sanchis et al. [92] who found that in older delirious ED patients, a higher level of chronicity was a predictor for mortality.

## Discussion

In this scoping review we identified a total of 114 publications providing an overview over the last decade of research regarding delirium in older patients in the ED setting. The current study included nine review publications and 105 original research studies, and most publications originated from North America and Europe. Through a team approach, we categorised all the publications into six main categories: *Prevention, Occurrence, Risk factors, Detection, Management and Consequences. Occurrence* (*n* = 55) and *Detection* (*n* = 50) had the largest number of publications, whereas *Prevention* had the smallest number (*n* = 10).

### Prevention

Prevention of delirium in older ED- patients were addressed in the fewest publications, despite evidence from other patient populations indicating that delirium is partially preventable. Consistent with our findings, a systematic review by Lee et al. [66] also only found four studies between 2010 and 2020 on delirium prevention or duration reduction in ED settings. Non-pharmacological, multicomponent interventions have demonstrated efficacy in reducing delirium incidence among hospitalized patients [131]. As studies on occurrence suggest, some patients present with delirium upon ED admission [31], making complete prevention of all delirium cases unfeasible. Nevertheless, targeted interventions in the ED may prevent delirium development for some patients and progression or worsening of symptoms in others. Notably, two recent studies [24, 25] reported that appropriate pain management in the ED may reduce delirium occurrence. Delirium prevention strategies was also one of the focuses of Kennedy et al. [23] developing a change package and toolkit to provide practical resources to support delirium care in the ED.

### Occurrence

Occurrence of delirium in older ED patients was assessed using both diagnostic standards (DSM-IV, DSM-V, ICD-10) and screening tools such as CAM. Considerable heterogeneity in study design, and variation in the chosen age groups, reflect the reported delirium occurrences. Advanced age is a well-established risk factor for delirium [1], and higher prevalence is thus expected in the oldest age groups, which may explain why some studies set the cut-off as high as 75 years. This underscores the importance of clearly defining the age group when reporting delirium occurrences, as prevalence estimates likely differs accordingly. Notably, some studies primarily included all adults aged ≥ 18 years [34, 55, 58, 88, 129]. While delirium risk increases with age and vulnerability, it is not exclusive to older adults; even previously healthy individuals may develop delirium in the context of severe illness or injury [1].

Importantly, variability in LOS in the ED prior to assessment ranged from <1 hour [44] <4 hours [31, 65] and up to 36 hours [69]. These differences may reflect global variations in ED practices, including whether short-stay unit beds are integrated within the ED [53], as well as factors such as overcrowding and boarding. Such methodological inconsistencies highlight the need for standardized approaches to defining age thresholds and timing of assessment to improve comparability and reliability of delirium prevalence estimates in ED research.

### Risk factors

A substantial proportion of the included publications examined risk factors for delirium in the ED. Dementia and cognitive impairment were among the most frequently reported patient-related factors, followed by frailty, limitations in activities of daily living (ADL), and sensory deprivation. The use of potentially inappropriate medications (PIMs), including opioids, benzodiazepines, and psychotropic drugs, was also identified as a significant contributor. The challenge of medication-related risk was highlighted in the 2024 systematic review of geriatric ED guidelines by Seidenfeld et al. [86]. Further, Raso et al. [53] reported a prevalence of 17.5% of older patients experiencing medication-induced hyperactive delirium in an ED attached ward. This underscores the importance of timely and accurate access to medical history and medication charts in the ED setting.

The study by Émond et al. [72] found that patients with ED associated delirium received more opioids in the ED. However, ED-pharmacological risk should be interpreted alongside studies addressing medication use for delirium management, particularly opioids. Daoust et al. [78] demonstrated that pain itself—rather than opioid administration—was a key risk factor for delirium. This finding aligns with studies by Saito et al. [24] and Snapp et al. [25], who report that appropriate pain management in elderly ED patients with acute abdominal conditions and hip fractures, was associated with delirium prevention. Further research is needed to clarify the role of pain control and to identify safe and effective pharmacological strategies for managing hyperactive delirium and aggression in the ED. Studies designed to investigate whether medication use induce delirium in older ED are warranted.

The ability to identify individuals most vulnerable to delirium is fundamental for implementing effective preventive strategies and ensuring optimal management. Several included studies explored practical approaches to risk stratification in older ED patients. Notably, the recent Delphi study by Lafuente-Lafuente et al. [83] proposed an easy-to-use risk factor checklist for this purpose; however, its clinical utility still requires validation in future studies.

Addressable risk factors within the ED environment include LOS and boarding time. Most studies demonstrated that prolonged LOS was associated with increased delirium risk, including the review by e Silva et al. [87], who found that stays exceeding 10 hours significantly raised delirium frequency. In contrast, Evensen et al. [73] did not observe this association, possibly due to their shorter exposure threshold (>4 hours). Similarly, Joseph et al. [82] reported that ED boarding—representing unnecessary waiting time for ward admission—also increased delirium risk. Reducing time spent in the busy ED environment therefore appears to be a clinically relevant recommendation.

Additional factors that may increase the risk for delirium, include urinary catheterization. Although indicated for residual urine, one included study [73] demonstrated an association between permanent catheter placement and delirium. This may be explained by increased immobilization, itself a recognized risk factor. Collectively, these findings emphasize the need for multifaceted strategies addressing both patient-related and environmental risk factors to reduce delirium incidence in ED settings.

### Detection

Factors influencing delirium detection in the ED were frequently addressed in the included studies, with diagnostic accuracy reported for a range screening-tool. The sheer number of available instruments underscores challenge of consistent implementation in clinical practice. Qualitative studies exploring HCP´s perspectives highlight persistent barriers, even when validated, rapid-to-use tools are accessible. Furthermore, emphasis on staff education and systematic implementation of screening for older adults at risk was consistently noted, aligning with the recommendations of the Kennedy et al. toolkit [23]. Similarly, Seidenfeld et al. [86] called for more implementation-focused research to determine how delirium detection in EDs can be optimized.

### Management

Managing delirium in the ED is challenging due to a busy, uncontrolled environment and communication barriers [104]. Early screening and structured protocols are recommended to ensure timely interventions [95, 103]. Multimodal strategies—including staff education, computerized tools, and non-pharmacological measures like early mobilization, sleep improvement, and sensory support—are key components, though evidence of effectiveness is mixed [119, 124]. No medications have been identified as being able to reduce delirium severity, symptoms, or mortality, however they may cause harm. Pharmacological treatment is only advised when patients are posing a risk of harm for themselves or others [1], situations which may arise in the ED. Thus, pharmacological management requires clear guidance. With higher use of standardized order sets, lower doses of benzodiazepine and haloperidol for delirium treatment have been reported [122].

Identifying underlying causes and optimizing physiology remain critical components of delirium management. In the search for causes, Butcher et al. [118] reported that head CT offers limited diagnostic value in the absence of other neurological signs, emphasising the need for judicious use of imaging. Conversely, obtaining informant history provides valuable insights into a patients baseline cognitive function and medical background [33]. Encouraging need for next of kin or caregivers to accompany patients to the ED, might therefore represent a practical strategy to support accurate assessment.

When delirium is detected, adequate patient management is essential; however, this was among the least represented categories in the included publications. Considerable overlap was noted between interventions described for prevention and those for management, as similar strategies may serve both purposes depending on patient status. Despite this, the current review highlights a significant gap in research on delirium prevention and management specifically within the ED setting. Future studies should focus on developing and evaluating ED-specific protocols that integrate prevention and management strategies to ensure timely, standardized, and patient-centred care. Implementing the quality indicators proposed by Schnitker et al. [123] and Filiatreault et al. [120] may guide this process and support hospital managers in monitoring and improving the quality of care for older ED patients.

### Consequences

The negative consequences of delirium are well described [2, 132]. Included publications assessing consequences of ED delirium in this scoping review, found that this condition was associated with adverse outcomes for the patient, including clinical deterioration, during and after hospitalisation [125]. Regarding mortality, some studies identified an association between ED delirium and mortality [26, 52, 64], others did not [56], suggesting variability related to follow-up duration or patient characteristics. In addition, delirium was linked to greater healthcare resource use, such as prolonged hospital stays, higher ICU admission rates, and more frequent ED revisits, highlighting its systemic impact. Predictors of mortality, including chronicity of underlying conditions, further underscore the need for risk stratification in this population. These findings are in line with the recent systematic review of Tesfaye et al. [132] studying long term outcomes of delirium after hospital discharge. Overall, these findings emphasize the importance of early identification and management of delirium in the ED to mitigate both short- and long-term consequences for patients and healthcare systems.

### Strengths and limitations

A key strength of this scoping review is the comprehensive overview of the research field the past shy of 11 years, highlighting well-studied areas, and identifying gaps, particularly in prevention and management. Importantly, the review also addresses the limited evidence on how existing toolkits can be implemented in clinical practice.

The search strategy covered six major databases, and the review adhered to established scoping review guidelines, including dual independent screening and a team-based approach to data extraction, minimizing selection and extraction bias.

However, heterogeneity in ED settings, age groups and study designs- limits generalisability, and readers should consider the applicability of findings to their own context. Additionally, our decision to categorize all included publications may have resulted in the omission of relevant information that did not fit within our defined categories. Of importance, the quality of each study and publications in terms of methodology were not evaluated.

Most included studies originate from North America and Europe which further restricts global relevance. Additionally, valuable data may have been missed from excluded publications Where ED-specific findings were not separable from inpatient data.

## Conclusions

This scoping review synthesises the current body of research on various dimensions of delirium among older adults in the emergency department setting. It further identifies critical knowledge gaps related to strategies for preventing delirium onset and optimizing the management of patients experiencing delirium. While existing studies provide valuable insights into prevalence, risk factors, and clinical outcomes, including developed toolkits, significant gaps remain regarding effective strategies for prevention, optimal management and the use of described toolkits in clinical practice. These gaps underscore the need for targeted interventions and robust implementation research to improve recognition and care for this vulnerable patient population.

## Electronic supplementary material

Below is the link to the electronic supplementary material.

Supplementary Material 1

Supplementary Material 2

## Data Availability

All data generated or analysed during this study are included in this published article and its supplementary information files. The search strategies are also available in supplementary files.

## Abbreviations

ADL: Activities of daily living
CT: Computed tomography
ED: Emergency Department
LOS: Length of Stay
JBI: Joanna Bricks Institute
PRISMA-ScR-checklist: Preferred Reporting Items for Systematic reviews and Meta-Analyses extension for Scoping Reviews
DSM-V: Diagnostic and Statistical Manual of Mental Disorders, fifth edition
ICD-10: International classification of diseases and related health problems, 10th edition
HCP: Health care personnel
BCAM: Brief Confusion Assessment method
CAM: Confusion Assessment method
CAM ICU: Confusion Assessment method for the intensive care unit
FamCAM: Family Confusion Assessment method
Modified bCAM: Modified Brief Confusion Assessment method
NuDESC: Nursing Delirium Screening Scale
Nurse screen: ED Nurse Delirium Screening form4
AT: The 4 A´s Test (Alertnes, Abbreviated mental test, Attention, Acute change)
DTS: Delirium Triage Screen
BS- EEG: Bispectral EEG
RADAR: Recognizing Acute Delirium As part of your Routine (the 6th vital sign)
OTTAWA 3DY: what Day, what Date, spell worlD, Year
mCAM ED tool: Modified Confusion Assessment Method for the Emergency Department
Chart based CAM tool: Method for using CAM in a record review
MRAS S: The modified Richmond Agitation Sedation Scale
Chart DEL: Chart- based Delirium Identification Instrument
6-CIT: 6-Item Cognitive Impairment Test
MOTYB-12/ MOTYB-6: Reciting the months of year/6 months backwards
LUNCH: Spelling the word LUNCH backwards
DRS-R98: Revised Delirium rating Scale
RASS: Richmond Agitation Sedation Scale
Delirium index: Delirium severity tool
DOSS: Delirium Observation Screening Scale
MDAS: Memorial delirium assessment scale
OPTA: Oder persons Technical Assistants (OPTAs)
ICU: Intensive care unit
