# Supplementary material for: Delirium in older adults in the emergency department: a scoping review of current research and identified knowledge gaps
Source: BMC Emerg Med. 2026 Apr 18;26:201. doi: 10.1186/s12873-026-01582-z (PMC13397615; doi:10.1186/s12873-026-01582-z)
Supplement: Supplementary file 1 — Supplementary Material 1 [file 12873_2026_1582_MOESM1_ESM.docx]

**Ovid MEDLINE(R) ALL <1946 to July 09, 2024>**

| 1 | Delirium/ | 12874 |
| --- | --- | --- |
| 2 | (delir* adj2 (treatment or prognosis or detection or detecting or diagnosis or assessment or pathway or management or prevent* or predicti*)).ti,ab,kf. | 4488 |
| 3 | delirium.ti,ab,kf. | 22748 |
| 4 | ((emergency adj2 (department or ward or room)) or casualty department).ti,ab,kf. | 148837 |
| 5 | ((sudden adj2 (confusion* or disorient* or psychosis)) or (acute adj2 (confusion* or disorient* or psychosis))).ti,ab,kf. | 2946 |
| 6 | 1 or 2 or 3 | 24663 |
| 7 | 5 or 6 | 27183 |
| 8 | 4 and 7 | 895 |
| 9 | limit 8 to (yr="2000 -Current" and "all aged (65 and over)") | 391 |

<https://ovidsp.ovid.com/ovidweb.cgi?T=JS&NEWS=N&PAGE=main&SHAREDSEARCHID=3Hi0X0wAhTKL1YvQ6H9wUSUSFmGqKzuQMtXoD5duWszFXrxO2mcYlep0SCHdO6fUj>

**Embase <1974 to 2024 Week 27>**

| 1 | delirium/ | 38011 |
| --- | --- | --- |
| 2 | delirium.ti,ab,kf. | 34712 |
| 3 | (delir* adj2 (treatment or prognosis or detection or detecting or diagnosis or assessment or pathway or management or prevent* or predicti*)).ti,ab,kf. | 6738 |
| 4 | ((emergency adj2 (department or ward or room)) or casualty department).ti,ab,kf. | 238240 |
| 5 | ((sudden adj2 (confusion* or disorient* or psychosis)) or (acute adj2 (confusion* or disorient* or psychosis))).ti,ab,kf. | 4689 |
| 6 | 1 or 2 or 3 or 5 | 52318 |
| 7 | 4 and 6 | 2184 |
| 8 | limit 7 to ("remove medline records" and yr="2000 -Current" and aged <65+ years>) | 490 |

**Epistemonikos**

((delir* OR delirium) AND (emergency room OR emergency department OR emergency ward)) OR abstract:((delir* OR delirium) AND (emergency room OR emergency department OR emergency ward))

**Cochrane library**

| 1 | Mesh(legg til fra mesh søk liste) delirium | [1604] |
| --- | --- | --- |
| 2 | (delir* near/2 (treatment or prognosis or detection or detecting or diagnosis or assessment or pathway or management or prevent* or predicti*)) | [1386] |
| 3 | delirium | [6442] |
| 4 | ((sudden near/2 (confusion* or disorientat* or psychosis)) or (acute near/2 (confusion* or disorientat* or psychosis))) | [356] |
| 5 | #1 or #2 or #3 or #4 | [6812] |
| 6 | ((emergency near/2 (department or ward or room)) or casualty department) | [19830] |
| 7 | #5 and #6 | [229] |

WoS

[Web of Science Core Collection](https://www.webofscience.com/wos/woscc/summary/679cd17a-a7cb-4ff6-a912-15b1d5c34037-fa88dd68/relevance/1)

| 1 | (TI=(((emergency near/1 (department or ward or room)) or casualty department) )) OR AB=(((emergency near/1 (department or ward or room)) or casualty department) ) | 147,129 |
| --- | --- | --- |
| 2 | (TI=((delir* near/1 (treatment or prognosis or detection or detecting or diagnosis or assessment or pathway or management or prevent* or predicti*)).)) OR AB=(((delir* near/1 (treatment or prognosis or detection or detecting or diagnosis or assessment or pathway or management or prevent* or predicti*)).)) | 4,392 |
| 3 | ((TI=(((sudden near/1 (confusion* or disorientat* or psychosis)) or (acute near/1 (confusion* or disorientat* or psychosis))))) OR AB=(((sudden near/1 (confusion* or disorientat* or psychosis)) or (acute near/1 (confusion* or disorientat* or psychosis))))) OR ALL=(delirium) | 28,646 |
| 4 | 2 or 3 | 28,652 |
| 5 | 1 and 4 | 1,025 |
| 6 | TS=(elder* or eldest or frail* or geriatri* or "old age*" or "oldest old*" or senior* or senium or "very old*" or septuagenarian* or octagenarian* or octogenarian* or nonagenarian* or centarian* or centenarian* or supercentenarian* or "older people" or "older subject*" or "older patient*" or "older age*" or "older adult*" or "older man" or "older men" or "older male*" or "older woman" or "older women" or "older female*" or "older population*" or "older person*") | 2,294,471 |
| 7 | 5 and 6 | 662 |
| 8 | Refine year 2000-2024 and language English+Norwegian (othes not present) | 623 |

Psycinfo

<https://ovidsp.ovid.com/ovidweb.cgi?T=JS&NEWS=N&PAGE=main&SHAREDSEARCHID=4Gyql7nace6x8doZGm1GfbJrGSQsyedilCwJ9ds8U7EAsE9Li1qrS0us5QtBeM76K>

APA PsycInfo <1806 to July Week 1 2024>

| 1 | exp Delirium/ or exp psychosis/ or delir*.ti,ab,id. or ((sudden or acute) adj2 (confusion* or disorient* or psychosis)).ti,ab,id. or (delir* adj2 (treatment or prognosis or detection or detecting or diagnosis or assessment or pathway or management or prevent* or predicti*)).ti,ab,id. | 141036 |
| --- | --- | --- |
| 2 | ((emergency adj2 (department or ward or room)) or casualty department).ti,ab,id. | 14580 |
| 3 | exp Aging/ or exp Older Adulthood/ or "aging (attitudes toward)"/ or exp geriatrics/ or (elder* or eldest or frail* or geriatri* or old age* or oldest old* or senior* or senium or very old* or septuagenarian* or octagenarian* or octogenarian* or nonagenarian* or centarian* or centenarian* or supercentenarian* or older people or older subject* or older patient* or older age* or older adult* or older man or older men or older male* or older woman or older women or older female* or older population* or older person*).ti,ab,id. | 260958 |
| 4 | 1 and 2 and 3 | 102 |
| 5 | 1 and 2 | 725 |
| 6 | limit 5 to "380    aged <age 65 yrs and older>" | 158 |
| 7 | 4 or 6 | 186 |

CINAHL

| 1 | TI ( elder* or eldest or frail* or geriatri* or old age* or oldest old* or senior* or senium or very old* or septuagenarian* or octagenarian* or octogenarian* or nonagenarian* or centarian* or centenarian* or supercentenarian* or older people or older subject* or older patient* or older age* or older adult* or older man or older men or older male* or older woman or older women or older female* or older population* or older person* ) OR AB ( elder* or eldest or frail* or geriatri* or old age* or oldest old* or senior* or senium or very old* or septuagenarian* or octagenarian* or octogenarian* or nonagenarian* or centarian* or centenarian* or supercentenarian* or older people or older subject* or older patient* or older age* or older adult* or older man or older men or older male* or older woman or older women or older female* or older population* or older person* ) OR ( (MH "Aged, 80 and Over") OR (MH "Aged")  OR (MH “Geriatric Psychiatry”) ) | 1,069,312 |
| --- | --- | --- |
| 2 | (MH "Delirium") or delirium or (delir* N1 (treatment or prognosis or detection or detecting or diagnosis or assessment or pathway or management or prevent* or predicti*) OR (sudden or acute) n1 confusion* or disorient* or psychosis) | 30,270 |
| 3 | ((emergency N1 (department or ward or room)) or casualty department) | 77,055 |
| 4 | 1 and 2 and 3 | 359 |
| 5 | 2 and 3 Narrow by SubjectAge 65+ years | 280 |
